# Supplementary material for: SHADE: A multilevel Bayesian framework for modeling directional spatial interactions in tissue microenvironments
Source: PLoS Comput Biol. 2026 Feb 4;22(2):e1013930. doi: 10.1371/journal.pcbi.1013930 (PMC12890221; doi:10.1371/journal.pcbi.1013930)
Supplement: S1 Text — (PDF) [file pcbi.1013930.s001.pdf]

S1 Text. Supporting Information

Joel Eliason, Michele Peruzzi, Arvind Rao

A Background: Multitype Gibbs Point Process Models and Relationship to SHADE

A.1 Multitype Gibbs Point Process Models

Multitype Gibbs point process (MGPP) models provide a general framework for modeling spatial patterns where interactions between points influence their configuration [Baddeley et al., 2015, Moller and Waagepetersen, 2003]. Unlike inhomogeneous Poisson processes, Gibbs processes explicitly model dependencies between points via pairwise interaction potentials.

A.1.1 Mathematical Formulation

For a spatial point pattern $\mathbf{x} = \{x_1, \ldots, x_n\}$ with type marks $m_i \in \{1, \ldots, K\}$, a multitype Gibbs point process is characterized by its conditional intensity function:

$$\lambda_k(v \mid \mathbf{x}) = \beta_k \exp\left\{\sum_{x_i \in \mathbf{x}} \log \gamma_{m_i,k}(\|v - x_i\|)\right\}, \tag{1}$$

where $\beta_k > 0$ is the baseline intensity for type k and $\gamma_{j,k}(r)$ is the pairwise interaction function between types j and k at distance r. Values $\gamma_{j,k}(r) > 1$ indicate attraction, $\gamma_{j,k}(r) < 1$ indicate repulsion, and $\gamma_{j,k}(r) = 1$ indicate independence.

A.1.2 Key Limitation: Symmetry Assumption

A fundamental constraint of standard Gibbs point processes is the symmetry condition:

$$\gamma_{j,k}(r) = \gamma_{k,j}(r) \quad \text{for all types } j, k \text{ and distances } r. \tag{2}$$

This requirement ensures the joint density defines a valid probability measure.

Biological implication: MGPPs *cannot* distinguish directional spatial associations. If tumor cells cluster around blood vessels, but vessels are not preferentially located near tumors, a symmetric MGPP estimates only the average of these distinct directional effects—a severe limitation for studying directional biological processes.

In practice, MGPPs also typically assume parametric interaction functions (e.g., Strauss process with $\gamma_{j,k}(r) = \gamma_{j,k}\mathbb{I}(r \leq R)$) with fixed interaction radii. Inference for Gibbs models is challenging due to intractable normalizing constants, though the Berman-Turner logistic regression approximation [Berman and Turner, 1992] provides a computationally efficient alternative to MCMC over point configurations.

A.1.3 Hierarchical Gibbs Models: Asymmetry via Type Ordering

One approach to modeling asymmetric spatial interactions is to impose an ordering on cell types [Högmander and Särkkä, 1999]. In a hierarchical Gibbs model, types are indexed $1, 2, \ldots, K$ with the convention that type k may depend on types $1, \ldots, k-1$ but not on types $k+1, \ldots, K$. The conditional intensity for type k becomes:

$$\lambda_k(v \mid \mathbf{x}_{<k}) = \beta_k \exp\left\{\sum_{j<k} \sum_{x_i \in \mathbf{x}_j} \log \gamma_{j,k}(\|v - x_i\|)\right\}, \tag{3}$$

where $\mathbf{x}_{<k}$ denotes the configuration of all types preceding k in the ordering. This permits directional interactions: type j can influence type k (for $j < k$) without requiring symmetric reciprocal effects. For example, if vasculature is ordered before immune cells, the model can capture how vessel locations structure immune infiltration without requiring vessels to preferentially locate near immune cells.

Hierarchical Gibbs models in the literature typically employ parametric interaction functions with fixed radii and have focused on single-image estimation rather than multilevel data structures.

A.2 How SHADE Extends the MGPP Framework

SHADE builds on the conceptual foundation of MGPPs but introduces three key innovations:

A.2.1 Asymmetric (Directional) Interactions

Unlike MGPPs, which require $\gamma_{j,k}(r) = \gamma_{k,j}(r)$, SHADE explicitly models asymmetric spatial associations via directional spatial interaction curves:

$$\mathrm{SIC}_{A_k \to B}(s) = \sum_{p=1}^{P} \delta_{A_k}^{(p)} \phi_p(s), \tag{4}$$

where $\mathrm{SIC}_{A_k \to B}(s)$ quantifies how type-A_k source cells at distance s affect the log-intensity of type-B target cells. Critically, $\mathrm{SIC}_{A_k \to B}(s)$ and $\mathrm{SIC}_{B \to A_k}(s)$ are estimated independently, capturing directional processes like immune recruitment by tumors or structural constraints imposed by vasculature.

A.2.2 Flexible Basis Function Expansions

Rather than parametric forms with fixed interaction radii, SHADE uses smooth radial basis functions $\phi_p(s)$ (e.g., Gaussian or B-splines) with data-driven coefficients $\delta_{A_k}^{(p)}$. This yields smooth, adaptable interaction curves avoiding restrictive parametric assumptions while hierarchical priors prevent overfitting.

A.2.3 Multilevel Bayesian Hierarchical Structure

Standard MGPPs and hierarchical Gibbs models focus on single-image estimation. SHADE instead models spatial interactions across nested levels of biological organization:

$$\delta_{A_k}^{(m,p)} \sim \mathcal{N}(\gamma_{A_k}^{(n(m),p)}, \sigma^2_{\text{image},p}) \quad \text{(image-level)} \tag{5}$$

$$\gamma_{A_k}^{(n,p)} \sim \mathcal{N}(\psi_{A_k}^{(g(n),p)}, \sigma^2_{\text{patient},p}) \quad \text{(patient-level)} \tag{6}$$

$$\psi_{A_k}^{(g,p)} \sim \mathcal{N}(\mu_p, \sigma^2_{\text{cohort},p}) \quad \text{(cohort-level)} \tag{7}$$

This hierarchical Bayesian framework, implemented via the logistic approximation and Hamiltonian Monte Carlo/variational inference, enables borrowing strength across images within patients and patients within cohorts, full posterior inference on derived quantities (SICs) at any hierarchical level with simultaneous credible bands, cohort-level comparisons of spatial organization patterns between biological groups, and quantification of heterogeneity via variance parameters at each level. This provides interpretable, uncertainty-aware inference on biologically meaningful spatial interaction patterns across multiple scales.

A.3 Summary of Key Differences

Table A in S1 Text summarizes the key methodological differences between standard MGPP models, hierarchical Gibbs models, and the SHADE framework.

Together, these innovations enable SHADE to overcome fundamental limitations of existing point process methods for analyzing spatial organization in multiplexed tissue imaging data, where directional biological processes, smooth distance-dependent interactions, and hierarchical uncertainty quantification are essential.

Table A. Comparison of spatial point process modeling approaches

Feature	Symmetric MGPP	Hierarchical Gibbs	SHADE
**Interaction symmetry**	Symmetric: $\gamma_{j,k}(r) = \gamma_{k,j}(r)$	Asymmetric via type ordering	Asymmetric: independent $\mathrm{SIC}_{A\to B}(s)$
**Interaction function**	Parametric with fixed radii	Parametric with fixed radii	Flexible basis expansions
**Data structure**	Single image	Single image	Multilevel: image $\subset$ patient $\subset$ cohort
**Posterior inference**	Not typically Bayesian	Not typically Bayesian	Full posterior with credible bands, heterogeneity quantification

B Logistic Regression Approximation Details

Direct estimation of the Poisson likelihood in spatial point process models typically requires numerical integration over a fine spatial grid, which becomes computationally expensive and unstable in high-resolution images. To address this, we follow the logistic regression approximation introduced by Baddeley et al. [2014], which avoids spatial gridding by introducing a set of dummy points D sampled from a homogeneous Poisson process with known intensity λ_{dummy}.

We define the combined set of points $Y = X_B \cup D$, and assign binary labels:

$$I(v) = \begin{cases} 1, & v \in X_B \\ 0, & v \in D \end{cases} \tag{8}$$

The conditional probability that a point v is a true (observed) point of type B is given by:

$$P(I(v) = 1) = \frac{\lambda(v)}{\lambda(v) + \lambda_{\text{dummy}}}, \tag{9}$$

leading to the following logistic regression model:

$$\log \frac{P(I(v) = 1)}{P(I(v) = 0)} = \boldsymbol{z}^\top(v)\boldsymbol{\beta} + \sum_{k=1}^{K} \boldsymbol{q}_{A_k}^\top(v)\boldsymbol{\delta}_{A_k}^{(m)} - \log \lambda_{\text{dummy}}, \tag{10}$$

This approximation circumvents key computational challenges of direct Poisson modeling. In Poisson models, fine spatial discretization leads to large numbers of empty pixels, often resulting in singular design matrices and unstable inference [Baddeley et al., 2015]. The Hauck-Donner effect [Hauck and Donner, 1977] can further distort uncertainty estimates. By reframing the problem as a binary classification task over observed and dummy points, the logistic approximation enables scalable, stable inference of spatial interaction effects at the image level.

C Interpreting Example Spatial Interaction Curves

Fig A in S1 Text shows two example SICs representative of those estimated from real data. Type 2 cells (cyan) exhibit a strong positive association at short distances, consistent with clustering behavior observed in immune infiltration around tumor cells. In contrast, Type 1 cells (red) show negative association at close range, suggestive of exclusion zones, as might be induced by physical barriers or competitive interactions in the tissue microenvironment.

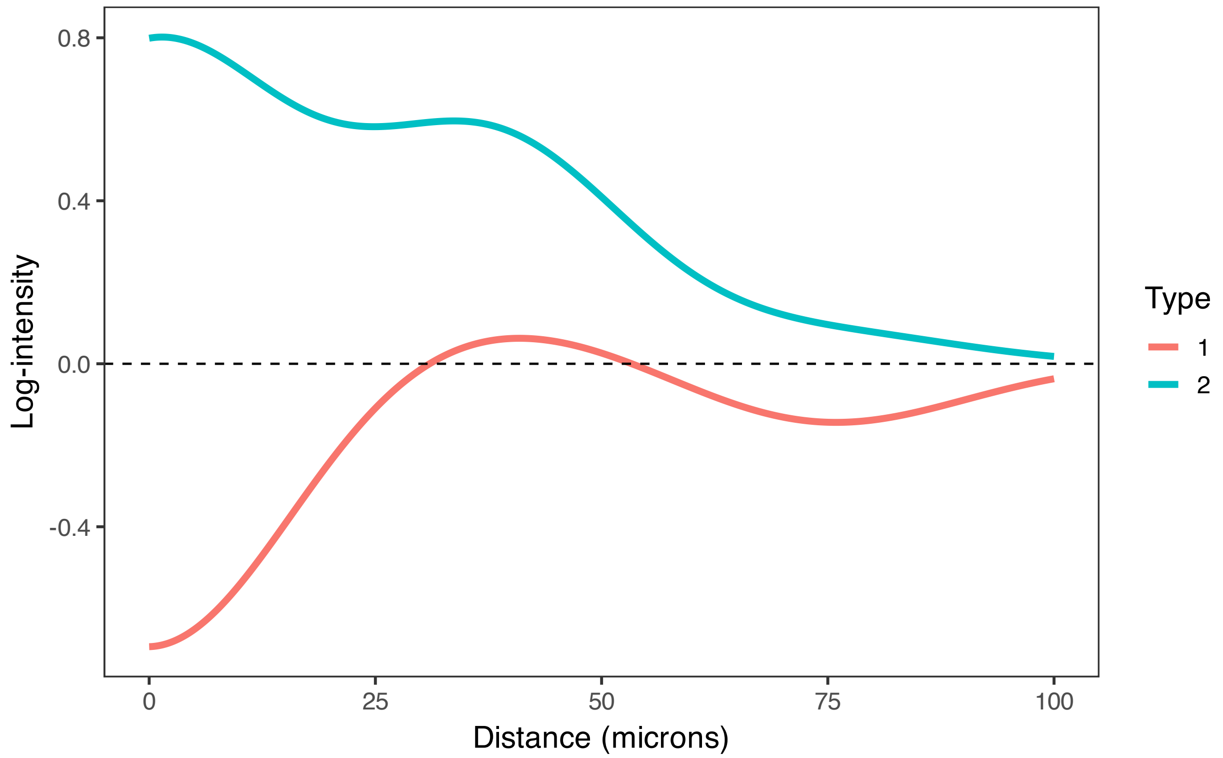

Fig A. Estimated SICs for two source cell types. Type 2 shows a strong positive association with the target population at short distances, while Type 1 exhibits negative association at short range. These contrasting patterns highlight the flexibility of the SIC framework for capturing biologically meaningful spatial structure.

Fig B in S1 Text demonstrates how these spatial interactions manifest in tissue space. Panels 1 and 2 show the spatial predictors $\boldsymbol{q}_{A_1}(v)$ and $\boldsymbol{q}_{A_2}(v)$ generated by the individual SICs from Fig A in S1 Text—each reflecting the localized contribution to the log-intensity of type B due to each source. Each panel is analogous to a summand from the sum on the right hand side of (2). Panel 3 shows the additive combination of these effects, illustrating how multiple source cell types jointly shape a heterogeneous spatial intensity landscape. The target cells are observed to cluster in high-intensity regions and avoid areas of low expected density.

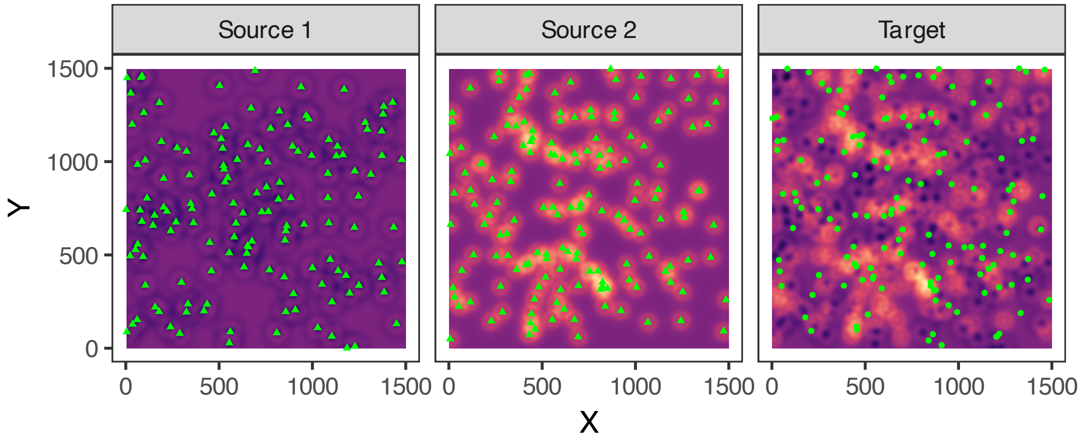

Fig B. Spatial interaction fields implied by SICs from two source cell types (Panels 1 and 2) and their additive combination (Panel 3). The combined field governs the spatial distribution of target cells, which are more likely to occur in regions of elevated log-intensity.

D Extended Methods and Results for Simulation Studies

D.1 Simultaneous Credible Bands for Spatial Interaction Curves

To quantify uncertainty in estimated spatial interaction curves (SICs), we constructed *simultaneous 95% credible bands* using posterior samples from our Bayesian model [Ruppert et al., 2003, Myllymäki et al., 2017]. While pointwise credible intervals can quantify uncertainty at individual distances, they do not account for multiple comparisons across the distance domain and tend to overstate confidence when evaluating the entire curve. In contrast, simultaneous credible bands provide a global probabilistic guarantee that the entire SIC lies within the band over its domain with high posterior probability.

Let $\widehat{\mathrm{SIC}}_{A_k \to B}(s)$ denote the posterior mean and $\hat{\sigma}_{A_k \to B}(s)$ the posterior standard deviation of the SIC at distance s, evaluated over a fixed grid of distances $s \in [r_{\min}, r_{\max}]$. For each posterior draw j, we computed the standardized residual

$$Z^{(j)}(s) = \frac{\mathrm{SIC}^{(j)}_{A_k \to B}(s) - \widehat{\mathrm{SIC}}_{A_k \to B}(s)}{\hat{\sigma}_{A_k \to B}(s)},$$

and then calculated the *maximum absolute standardized deviation* across the distance domain:

$$T^{(j)} = \sup_{s \in [r_{\min}, r_{\max}]} \left| Z^{(j)}(s) \right|.$$

This statistic captures the largest fluctuation, in units of local posterior uncertainty, that the sampled SIC exhibits relative to the posterior mean. By repeating this computation over posterior draws, we obtain the empirical distribution of the supremum deviation under the posterior. The critical value c for the

simultaneous band is then defined as the $(1-\alpha)$-quantile of the empirical distribution of $T^{(j)}$:

$$c = \text{quantile}_{1-\alpha}\left(\{T^{(1)}, \dots, T^{(J)}\}\right).$$

The resulting *simultaneous credible band* is:

$$\text{SIC}_{A_k \to B}(s) \in \left[\widehat{\text{SIC}}_{A_k \to B}(s) \pm c \cdot \hat{\sigma}_{A_k \to B}(s)\right], \quad \text{for all } s \in [r_{\min}, r_{\max}].$$

This construction ensures that with posterior probability $1-\alpha$, the entire SIC remains within the band across the full distance domain. It provides a conservative yet interpretable summary of global uncertainty, correcting for the multiple comparisons that arise when interpreting the SIC across many distances.

Simultaneous credible bands are particularly useful for assessing whether a spatial interaction is consistently positive, negative, or null over specific distance ranges, and for identifying features such as consistent attraction or repulsion that are unlikely to be due to noise. They also serve as a diagnostic for the informativeness of the data: wider bands reflect higher posterior uncertainty, particularly in regions where data are sparse or spatial patterns are weak.

All figures in the main manuscript showing SICs display simultaneous 95% credible bands computed via this method.

D.2 Screening Strategies for Multiple Cell Type Pairs

In exploratory spatial analyses involving many cell type pairs, we often wish to prioritize pairs for further investigation based on the estimated SICs and their uncertainty. While there are many ways to summarize spatial associations for this purpose, we propose three complementary summary measures based on simultaneous credible bands that address different biological questions:

D.2.1 Peak Location and Magnitude

To identify where the strongest interaction occurs, we locate the distance at which the SIC reaches its maximum absolute value, along with the magnitude at that location. Formally, for a given source–target pair $(A_k \to B)$, define:

$$s^* = \underset{s}{\text{argmax}}\, |\widehat{\text{SIC}}_{A_k \to B}(s)|, \quad M = |\widehat{\text{SIC}}_{A_k \to B}(s^*)|,$$

and assess statistical significance by checking whether the simultaneous credible band excludes zero at s^*. This identifies the distance of maximal association and its strength, and is particularly useful for identifying

pairs with strong localized effects—for example, immune cells that show pronounced clustering around tumor cells at a specific distance range.

D.2.2 Persistence Over Biologically Relevant Distance Ranges

To assess whether an interaction is consistently positive or negative over a pre-specified biologically meaningful distance range $I = [s_a, s_b]$, we compute the posterior probability that the SIC maintains a consistent sign throughout the interval. For each posterior draw j, we evaluate the SIC at all distances within I and record whether the entire curve is positive (or negative):

$$\Pi_I^{(+)} = \frac{1}{J}\sum_{j=1}^{J} \mathbb{I}\left\{\min_{s\in I} \mathrm{SIC}^{(j)}_{A_k\to B}(s) > 0\right\}, \quad \Pi_I^{(-)} = \frac{1}{J}\sum_{j=1}^{J} \mathbb{I}\left\{\max_{s\in I} \mathrm{SIC}^{(j)}_{A_k\to B}(s) < 0\right\}.$$

We then define $\Pi_I = \max(\Pi_I^{(+)}, \Pi_I^{(-)})$ as the persistence score. Values $\Pi_I \geq 0.95$ indicate strong evidence for persistent directional association throughout the interval—that is, robust associations that are unlikely to be artifacts of noise or multiple testing. For instance, in tumor immunology, one might define $I = [10, 50]$ μm to capture local microenvironmental interactions.

D.2.3 Overall Strength

To quantify the cumulative magnitude of a spatial interaction across all distances where it is statistically significant, we integrate the absolute effect size over significant regions:

$$\mathrm{Strength}(A_k \to B) = \int_{I_{\mathrm{sig}}} |\widehat{\mathrm{SIC}}_{A_k\to B}(s)|\, ds,$$

where I_{sig} is the union of all intervals where the simultaneous credible band excludes zero. This captures cumulative association strength and provides a single summary of the overall importance of a spatial interaction. Cell type pairs with high strength scores exhibit either strong localized effects or more moderate effects that persist across many distances.

D.2.4 Usage in Practice

These three measures can be computed across all source–target pairs and visualized as heatmaps to facilitate systematic comparison. Peak location and magnitude would require either two separate heatmaps (one for s^*, one for M) or a combined visualization (e.g., color-coding magnitude with symbol size indicating distance). The persistence and overall strength measures each yield a single scalar value per pair and map directly to heatmap intensities. These visualizations allow pairs to be ranked and prioritized for detailed examination.

The choice among these measures depends on the biological question: peak magnitude for identifying strongest interactions, persistence for testing specific distance-based hypotheses, or overall strength for comparing cumulative effects. They are complementary and can be used together to provide multiple perspectives on spatial organization patterns.

D.3 Extended Methods for Hyperparameter Studies and Study of Explicit Modeling of Multilevel Structure

To evaluate SHADE's performance under controlled conditions and assess the effect of modeling multilevel structure, we conducted simulation studies generating synthetic spatial point patterns with known hierarchical structure and asymmetric spatial interactions. This section describes the data generation process and default parameter settings used across simulation experiments, including: (1) comparison of hierarchical vs. non-hierarchical models (Section D.4 in S1 Text), and (2) hyperparameter studies examining the effects of cell count, dummy point ratio, and dataset size (Section D.6 in S1 Text and Section D.7 in S1 Text).

The simulation process consists of three steps: (1) generating hierarchical spatial interaction coefficients according to the model in Equation 5, (2) generating source cell locations from a homogeneous Poisson process, and (3) generating target cell locations from an inhomogeneous Poisson process whose intensity depends on proximity to source cells via spatial interaction features.

D.3.1 Generating Target Points

Given source cell locations and hierarchical spatial interaction coefficients, target cell locations are drawn from an inhomogeneous Poisson process with conditional intensity:

$$\lambda(v) = \exp\left(\sum_{k=1}^{K}\sum_{p=1}^{P} \boldsymbol{q}_{A_k}^\top(v)\boldsymbol{\delta}_{A_k}^{(m)} + \beta_0^{(m)}\right), \tag{11}$$

where $\beta_0^{(m)}$ is a normalization offset to ensure that the expected number of target points matches N_{points} in image m. It is computed as:

$$\beta_0^{(m)} = \log\left(\frac{N_{\text{points}}}{\int_W \exp\left(\sum_{k=1}^{K}\sum_{p=1}^{P} \boldsymbol{q}_{A_k}^\top(v)\boldsymbol{\delta}_{A_k}^{(m)}\right) dv}\right). \tag{12}$$

D.3.2 Default Parameter Settings

Unless otherwise specified, the following defaults were used in simulation experiments:

- **Spatial domain:** $S = 1500$.

- **Number of source cell types:** $K = 2$.
- **Basis functions:** $P = 3$ radial basis functions with bandwidth 15 and support up to 75 microns.
- **Points per cell type per image:** $N_{\text{points}} = 150$.
- **Variance parameters:**
  - $\sigma_{\text{cohort},p} = 0.5$
  - $\sigma_{\text{patient},p} = 0.1$
  - $\sigma_{\text{image},p} = 0.1$
- **Number of patients and images:** variable across experiments.

D.4 Results: The effect of explicitly modeling multilevel structure

We examined how accounting for hierarchical structure affects inference quality by comparing the full hierarchical model against a non-hierarchical alternative that estimates image-level SICs independently, without shrinkage to patient- or cohort-level structures. We simulated data from two patient groups, each with 20 patients and 4 images per patient. Each image contained 150 points of each type, with a dummy-to-real point ratio of 2. Remaining parameters followed the defaults specified in Section D.3.2 in S1 Text.

We generated 100 simulation replicates and fit both models to each replicate. Inference quality was assessed by comparing the average RMSE of the $\delta_{t_1 \to t_2}^{(m,p)}$ coefficients.

We found that on average, and across all spatial scales, the average RMSE of $\delta_{t_1 \to t_2}^{(m,p)}$ coefficients was lower for those estimated by the full hierarchical model than the model that fit images individually, most significantly so for closer-range coefficients (Table B in S1 Text).

Hierarchical	Scale: All	Scale: Small	Scale: Medium	Scale: Large
False	0.142 (0.097, 0.196)	0.355 (0.230, 0.509)	0.022 (0.016, 0.031)	0.048 (0.034, 0.066)
True	0.039 (0.027, 0.043)	0.072 (0.043, 0.084)	0.017 (0.013, 0.020)	0.028 (0.021, 0.036)

Table B. Average RMSE of $\delta_{t_1 \to t_2}^{(m,p)}$ coefficients in aggregate and across spatial scales, comparing hierarchical and non-hierarchical models.

Fig C in S1 Text shows several examples of image-level SICs estimated with both models. In all cases, the SIC is better estimated when hierarchical structure is accounted for, with improvements in both bias and variance. Table B in S1 Text quantifies this improvement: the hierarchical model achieves substantially lower RMSE across all spatial scales, with the most dramatic improvement at small scales (RMSE reduction from 0.355 to 0.072).

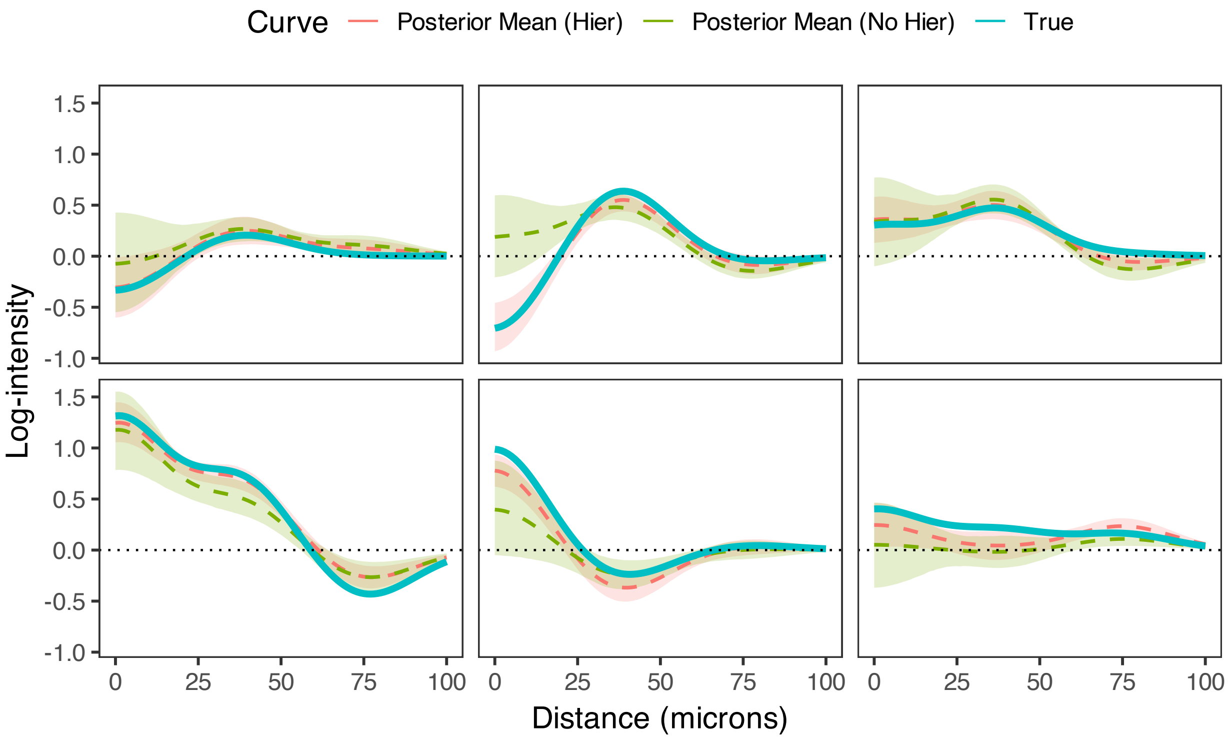

Fig C. Examples of estimated image-level SICs from hierarchical modeling simulations, with simultaneous 95% credible bands, demonstrating better estimation of SICs when accounting for multilevel structure.

D.5 Extended Methods for Comparison of Spatial Pattern Detection Accuracy Across Methods and Conditions

To evaluate SHADE's detection capabilities relative to standard spatial analysis methods and assess how performance varies with data characteristics, we conducted a factorial simulation study varying cell densities and number of images per patient. All simulated datasets contained a single patient cohort with known positive spatial interactions (attraction pattern) at multiple distance scales. This section describes the data generation process, experimental design, and evaluation metrics for the method comparison study reported in Section 3.1 of the main text.

D.5.1 Hierarchical Data Generation

We simulate spatial interaction coefficients $\delta^{(m,p)}$ at three hierarchical levels (for image m and basis function $p \in \{1, 2, 3\}$) for a single patient cohort:

$$\psi^{(p)} = (1.5,\ 1.0,\ 0.5) \quad \text{for } p \in \{1,2,3\} \quad \text{(cohort-level, fixed)}$$
$$\gamma^{(n,p)} \sim \mathcal{N}(\psi^{(p)},\ \sigma^2_{\text{patient}}), \quad \sigma_{\text{patient}} = 0.1$$
$$\delta^{(m,p)} \sim \mathcal{N}(\gamma^{(n(m),p)},\ \sigma^2_{\text{image}}), \quad \sigma_{\text{image}} = 0.1$$

where $n(m)$ denotes the patient corresponding to image m. The positive cohort-level coefficients create an attraction pattern between source and target cells across all distance scales, with the strongest effect at short range ($\psi^{(1)} = 1.5$) and decreasing effects at medium ($\psi^{(2)} = 1.0$) and long range ($\psi^{(3)} = 0.5$).

D.5.2 Spatial Pattern Generation

Cell patterns were generated using spatially varying intensity functions within $1500 \times 1500\ \mu\text{m}^2$ observation windows. T cells and B cells were distributed as independent Poisson processes with densities λ_T and λ_B. Tumor cell locations were generated using a spatially varying intensity surface based on T cell locations:

$$\lambda_{\text{tumor}}(s; n) = \exp\left(\beta_0 + \sum_p \delta^{(n,p)} \phi_p(s)\right)$$

where $\phi_p(s)$ represents radial basis functions with $\sigma = 15\ \mu$m and centered at $\mu = (0, 40, 80)\mu m$. The baseline intensity β_0 was calibrated to achieve target tumor cell densities, as in (12) in S1 Text.

D.5.3 Experimental Design

We employed a $2 \times 2 \times 3$ factorial design varying the following factors:

- **T cell (source) density:** High (150 cells) vs. Low (15 cells) per image.
- **Tumor cell (target) density:** High (150 cells) vs. Low (15 cells) per image.
- **Images per patient:** 1, 2, or 3 tissue sections.
- **Replication:** 50 independent simulations per condition.

All simulations included 40 patients in a single cohort and an additional B cell population (density matched to T cells) as a third cell type. This design yielded 12 experimental conditions ($2 \times 2 \times 3$) with 50 replicates each (600 total simulations), allowing systematic assessment of how detection power and calibration vary with data quantity and hierarchical structure.

D.5.4 Evaluation Metrics

SHADE Performance: We assessed SHADE's ability to recover true spatial interaction curves by evaluating whether 95% simultaneous credible bands (Section D.1 in S1 Text) correctly identified the known positive spatial interaction (attraction pattern). Detection success required the credible band to exclude zero at any distance in the 0–75 μm range.

Envelope Test Comparisons: We compared SHADE against G-cross and L-cross envelope tests implemented using the `spatstat` package. Global envelopes were constructed using 99 Monte Carlo simulations of point patterns generated according to complete spatial randomness (CSR). The rank parameters were calibrated in preliminary null scenario simulations to achieve proper Type I error control: `nrank = 10` for G-cross (yielding nominal significance level $\alpha = 10/100 = 0.10$) and `nrank = 8` for L-cross (yielding $\alpha = 8/100 = 0.08$). These nominal levels achieved empirical Type I error rates of 2.5–8.8% for G-cross and 1.3–6.7% for L-cross across different density regimes (Section D.9 in S1 Text). Detection success required the observed summary function to deviate outside the simultaneous envelope at any distance, indicating rejection of the null hypothesis of complete spatial randomness.

Statistical Analysis: Power was calculated as the average over all images of the proportion of correctly classified interactions at target distances. We compared both methods' sensitivity to varying cell densities and sample sizes. Additionally, we evaluated coverage (the proportion of true SIC values falling within the 95% simultaneous credible bands) and type I error rates (the proportion of null interactions incorrectly identified as significant) for all methods.

D.6 Hyperparameter Study 1 - The effect of cell count and dummy point ratio on inference quality

We investigated how inference quality is affected by the number of observed cells per cell type (N_t) and the ratio of dummy points to real points (R_d) used for quadrature. Simulations were run for all combinations of $N_t \in \{20, 80, 150, 300\}$ and $R_d \in \{0.5, 1, 2, 5, 10\}$, with five replicates per setting. Each dataset contained three cell types, with the third serving as the target for spatial interaction estimation. The number of patient groups was fixed at 1, with 40 patients per group and two images per patient. All other simulation parameters were set to defaults.

Average root mean squared error (RMSE) was computed for spatial interaction coefficients at the image ($\delta^{(m,p)}_{t_1 \to t_2}$), patient ($\gamma^{(n,p)}_{t_1 \to t_2}$), and cohort ($\psi^{(g,p)}_{t_1 \to t_2}$) levels. As shown in Fig D in S1 Text, RMSE for image-level coefficients decreased consistently with increasing R_d, suggesting that finer quadrature grids improve inference at the image level. This trend held across all values of N_t.

In contrast, inference quality for patient- and cohort-level coefficients slightly worsened when R_d exceeded 2–5, particularly at very low or very high values of N_t. This suggests diminishing returns—and potential instability—for higher-level parameter estimation when dummy point counts are excessively large. The magnitude of this effect was modest but visible (note the log scale on the RMSE axis).

To further investigate these trends, we analyzed RMSE stratified by spatial scale (short, medium, long) at each hierarchical level (Fig E in S1 Text). Improvements in image-level inference with increasing R_d were most pronounced at longer interaction distances. For higher-level parameters, performance remained relatively stable across spatial scales but showed a slight increase in RMSE at short ranges when dummy point ratios were high.

A representative cohort-level SIC estimate is shown in Fig F in S1 Text, illustrating how estimation accuracy varies with N_t and R_d. Inference was notably poorer at low cell counts, with increased bias and wider credible intervals. Optimal performance—reflected in reduced bias and uncertainty—was observed at moderate N_t (80–300) and a wide range of dummy point ratios, consistent with the RMSE patterns observed in Fig D in S1 Text.

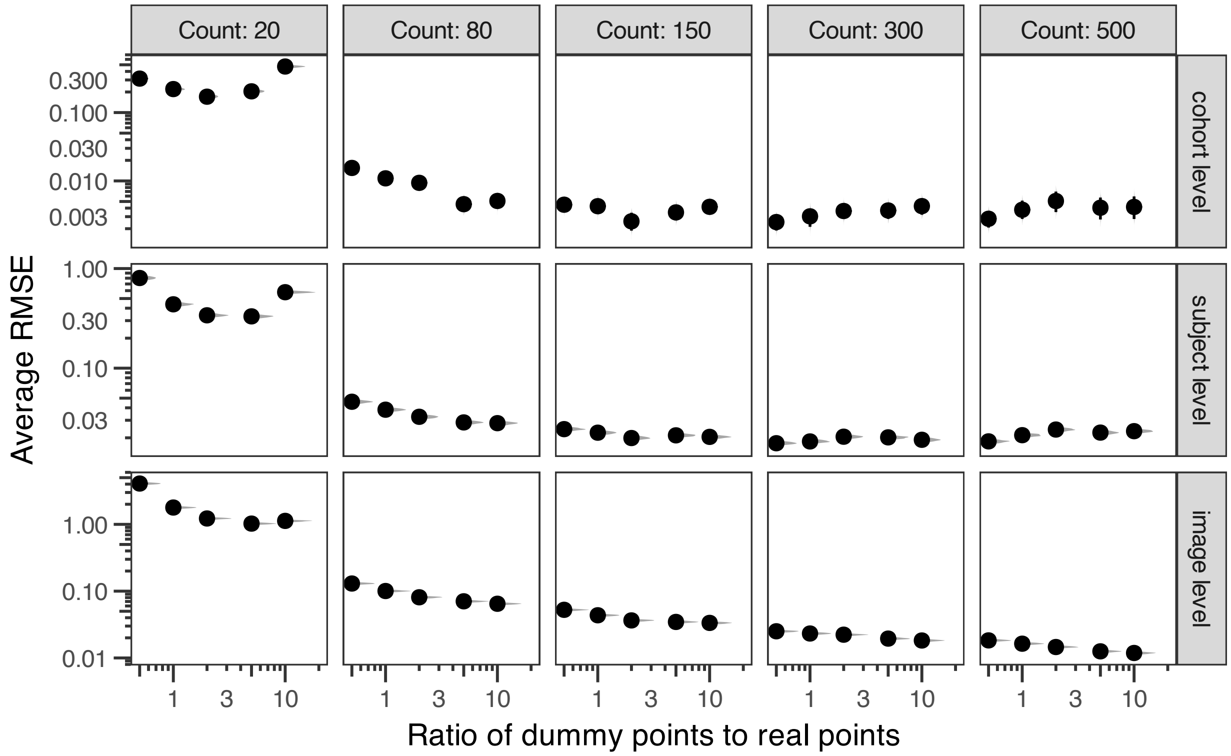

Fig D. Average RMSE for spatial interaction coefficients across hierarchical levels: image-level ($\delta^{(m,p)}_{t_1 \to t_2}$), patient-level ($\gamma^{(n,p)}_{t_1 \to t_2}$), and cohort-level ($\psi^{(g,p)}_{t_1 \to t_2}$), under different numbers of cells per type and dummy-to-real point ratios.

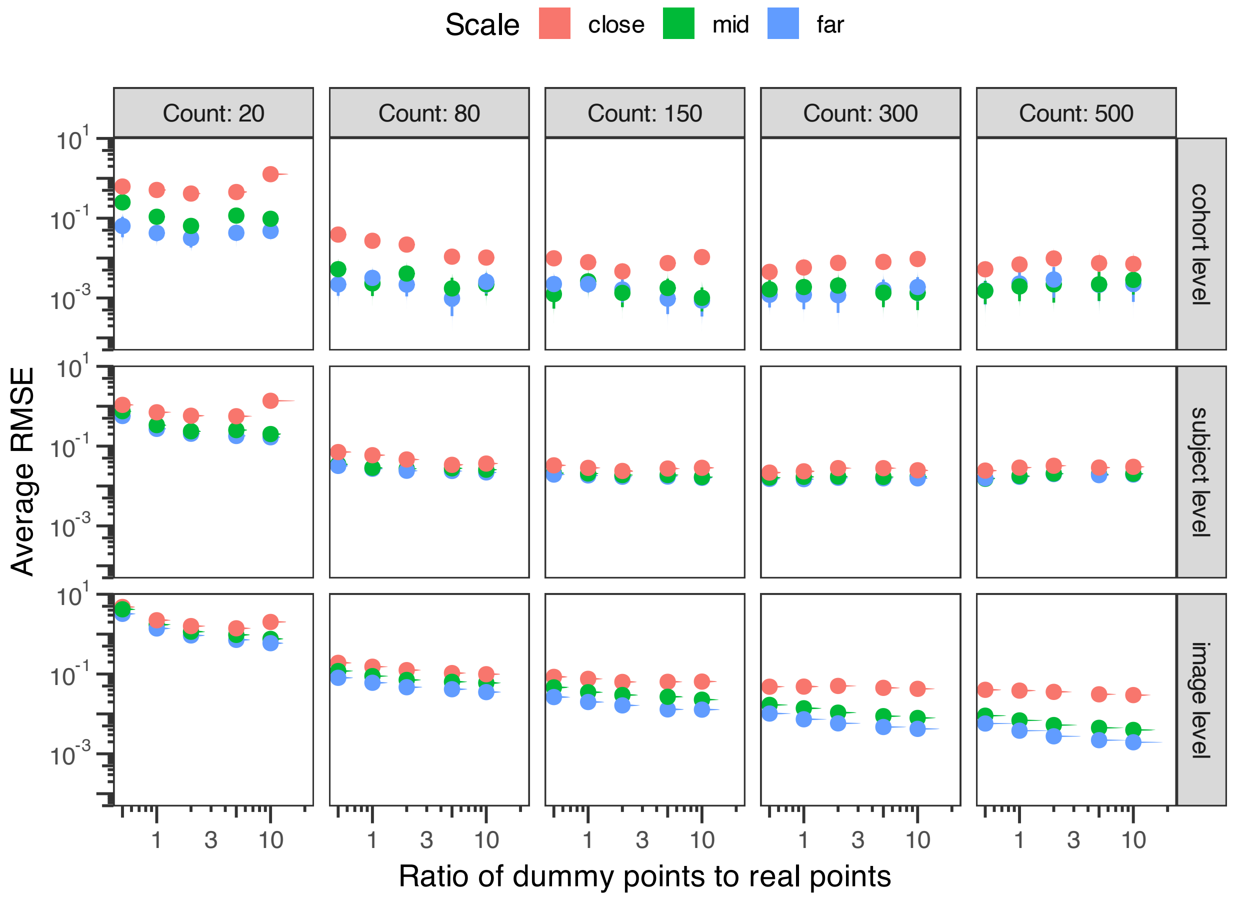

Fig E. RMSE for spatial interaction coefficients across spatial scales, shown separately for each hierarchical level: $\delta^{(m,p)}_{t_1\to t_2}$, $\gamma^{(n,p)}_{t_1\to t_2}$, and $\psi^{(g,p)}_{t_1\to t_2}$. Results are shown for varying cell counts and dummy point ratios.

D.7 Hyperparameter Study 2 - The effect of number of patients and images per patient on inference quality

Next, we examined the sensitivity of inference quality to the total number of patients as well as the number of images per patient. Here, we set the number of points per type to 150 and the ratio of dummy points to actual points to 2, while keeping the rest of the default parameters the same, as outlined in Section D.3.2 in S1 Text. We then simulated 15 realizations each of every combination of the number of images per patient $N_{\text{images per patient}}$ and the total number of patients N_{patients}, where $N_{\text{images per patient}} \in \{1, 2, 4\}$ and $N_{\text{patients}} \in \{10, 20, 40\}$, according to our simulation procedure described in Section D.5 in S1 Text.

We found some interesting trends in the average RMSE as the number of patients per patient group increased. For cohorts that only had one image per patient, average RMSE decreased as the number of patients increased (Figure G). However, for patients that had 2 images, the average RMSE was highest for the highest number of patients, which we found to be a counterintuitive finding. Furthermore, for patients

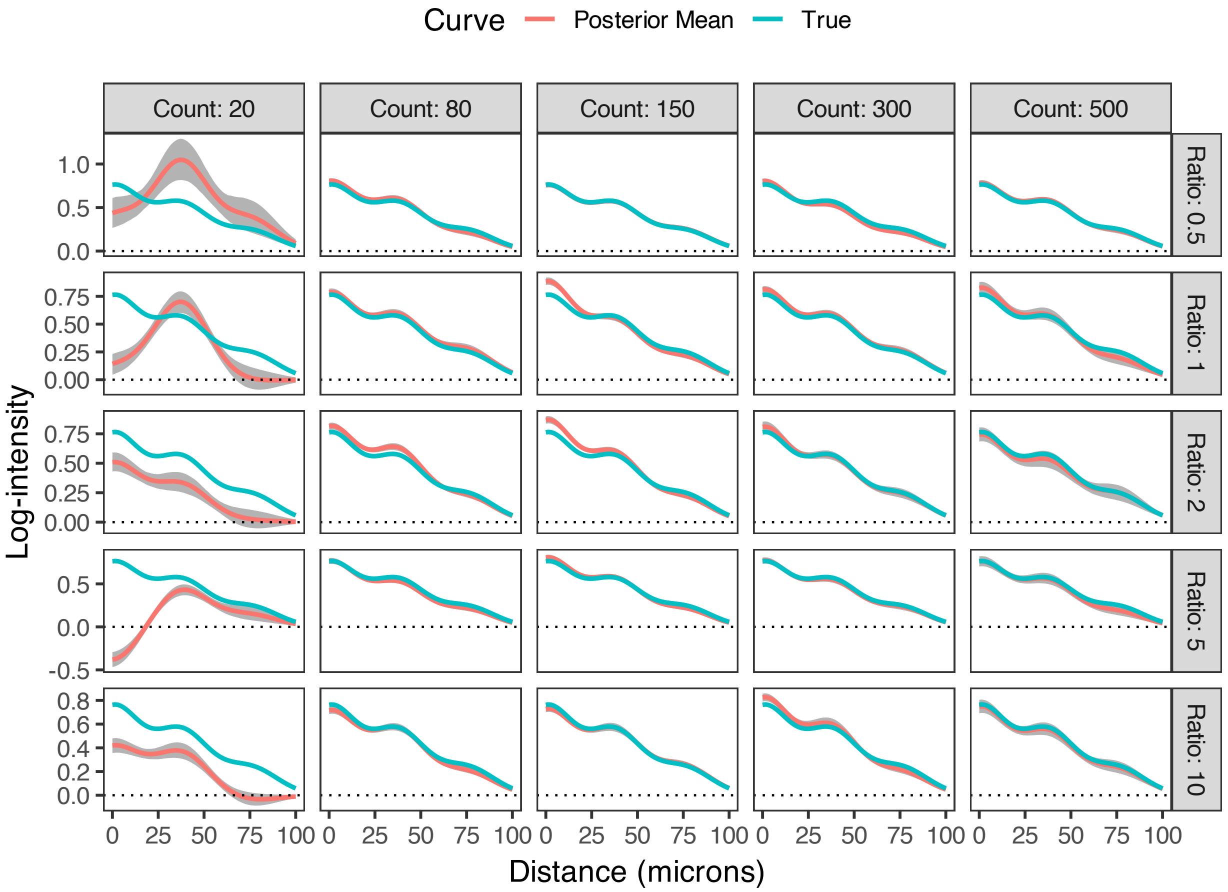

Fig F. Example of estimated cohort-level SIC from dummy point simulations.

with 4 images, having a cohort with 40 patients was associated with the lowest RMSE, as expected, though the second-lowest was, unexpectedly, having a cohort with 10 patients, rather than 20.

For $\delta^{(m,p)}_{t_1 \to t_2}$ coefficients, longer-range coefficients seemed to have the lowest average RMSE (Fig H in S1 Text), while close-range coefficients had the worst quality. RMSE, however, was very close to constant across the number of patients, though it did decrease slightly as the number of images increased. RMSE for $\gamma^{(n,p)}_{t_1 \to t_2}$ coefficients decreased as the number of images increased, though, as we noticed earlier, the RMSE in this case has a nonlinear relationship with the number of patients. The RMSE of $\psi^{(g,p)}_{t_1 \to t_2}$ coefficients exhibited the same counterintuitive nonlinear association with number of patients, though in a more pronounced way.

Finally, we demonstrate an example of an estimated global SIC from this simulation study (Figure I). Here, we can see that the variance of the SIC estimate decreases both as the number of patients increases and the the number of images increases, which matches our expectations. However, we can also see that there is a slight amount of persistent bias at close range for simulations in which the number of patients equals 40 - this may be indicative of too much shrinkage during estimation and may be the reason for the

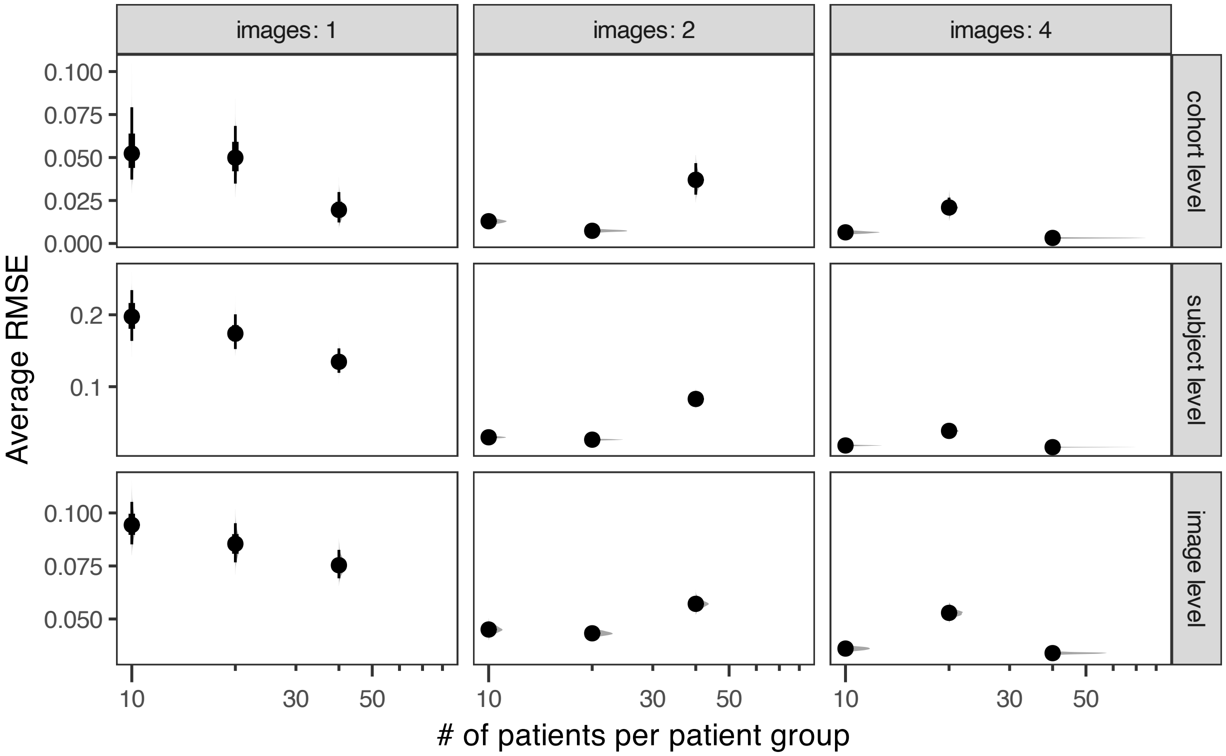

Fig G. Average RMSE for spatial interaction coefficients as a function of dataset size, varying the number of patients and number of images per patient. Results are shown for image-level ($\delta^{(m,p)}_{t_1\to t_2}$), patient-level ($\gamma^{(n,p)}_{t_1\to t_2}$), and cohort-level ($\psi^{(g,p)}_{t_1\to t_2}$) parameters.

nonlinear association of RMSE with number of patients.

D.8 Computational Scaling: Timing Experiments

To assess the practical computational feasibility of SHADE for large-scale spatial datasets, we conducted timing experiments measuring both feature construction time and full model fitting time across simulated datasets ranging from 5,000 to 250,000 cells.

D.8.1 Methods

We generated synthetic spatial point patterns with 3 cell types (2 source types, 1 target type) across six cell count conditions: 5,000, 10,000, 25,000, 50,000, 100,000, and 250,000 total cells. To reflect realistic experimental designs, we used a hierarchical structure with 40 patients and 4 images per patient (160 total images), with total cell counts distributed across all images. For each condition, we simulated 20 independent replicates using a fixed spatial window (1500 × 1500 units) per image, so that cell density increased proportionally with cell count. Each pattern was generated with spatial interactions defined by

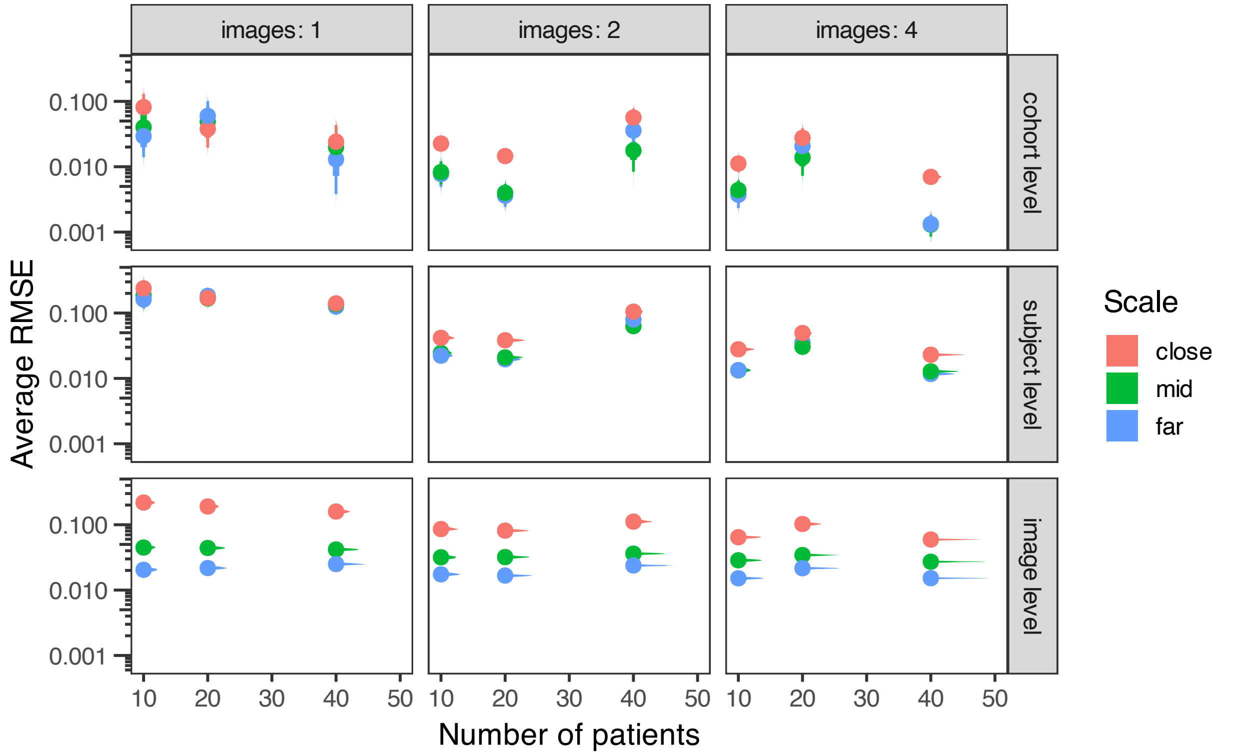

Fig H. Average RMSE across spatial scales for spatial interaction coefficients at the image ($\delta_{t_1 \to t_2}^{(m,p)}$), patient ($\gamma_{t_1 \to t_2}^{(n,p)}$), and cohort ($\psi_{t_1 \to t_2}^{(g,p)}$) levels, across varying numbers of patients and images per patient.

three radial basis functions, matching the setup used in our main simulations.

For each replicate, we measured two key computational steps:

1. **Feature construction time**: The time required to compute pairwise distances between target and source cells and construct the interaction feature matrix $\boldsymbol{q}_{A_k}(v)$ for all spatial locations (observed cells and dummy points). This step involves distance matrix computation via `spatstat.geom::crossdist` followed by basis function evaluation and summation.

2. **Total model fitting time**: The end-to-end time from loading the prepared data to obtaining posterior draws from the fitted SHADE model. Models were fit using variational inference with 1,000 posterior draws on a single CPU core (no parallelization).

All timing measurements were performed on a high-performance computing cluster with consistent hardware specifications to ensure comparability across conditions. Timing was recorded using the `tictoc` R package.

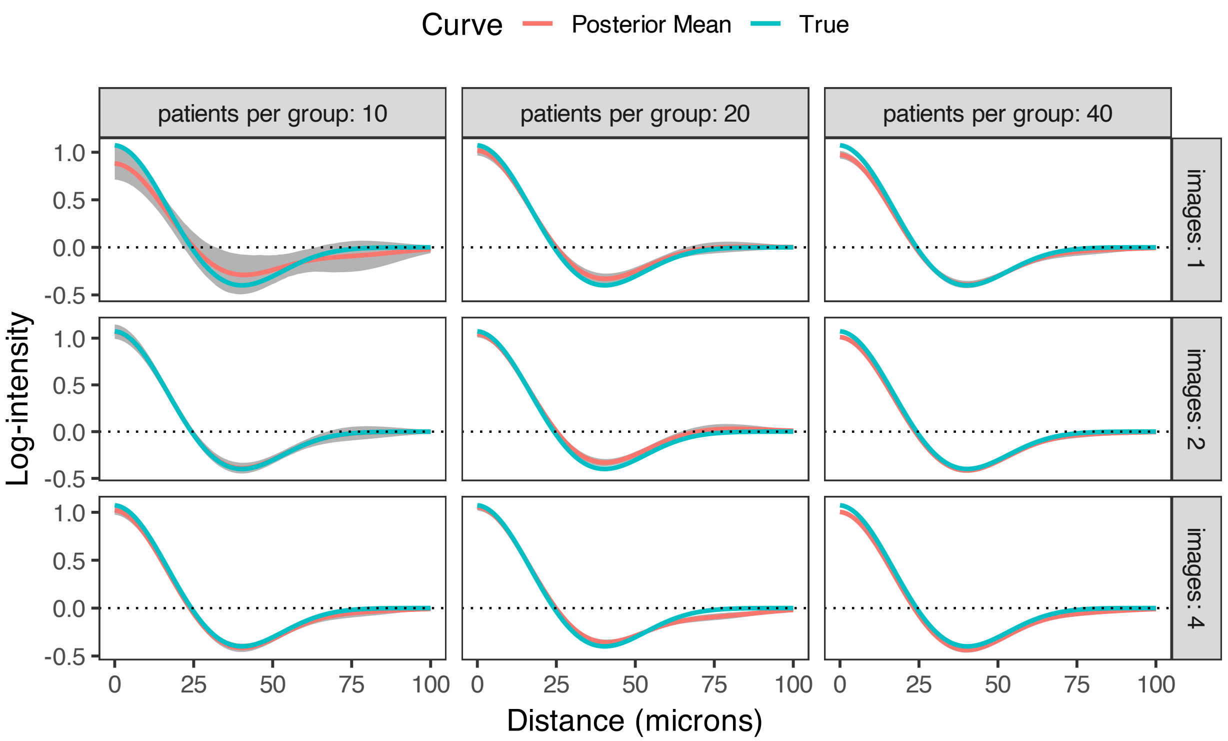

Fig I. Example of an estimated cohort-level SIC from the dataset size simulation study, illustrating how increasing the number of patients and images per patient reduces the variance in SIC estimates.

D.8.2 Results

Fig J in S1 Text shows the scaling behavior of feature construction and model fitting times as a function of the total number of cells. Feature construction time exhibited near-quadratic scaling with an empirical exponent of 1.46 (95% CI from log-log regression), consistent with the $\mathcal{O}(n_{\text{target}} \times n_{\text{source}})$ complexity of computing pairwise distances. Total model fitting time scaled sublinearly with an empirical exponent of 0.85, substantially better than the naive quadratic expectation, due to the efficiency of the variational inference algorithm and reuse of the distance matrix across all basis functions and source types.

These results demonstrate that SHADE remains computationally tractable even for large-scale datasets. At 100,000 cells—substantially larger than most contemporary spatial profiling datasets—mean total fitting time was 36.4 seconds (±4.1 SD). Even at 250,000 cells, mean fitting time remained 133.1 seconds (±46.2 SD), on the order of minutes rather than hours, making SHADE practical for routine application to large-scale tissue imaging data. Feature construction accounted for approximately 9.1 seconds (100K cells) and 37.3 seconds (250K cells), representing roughly 25–28% of total runtime.

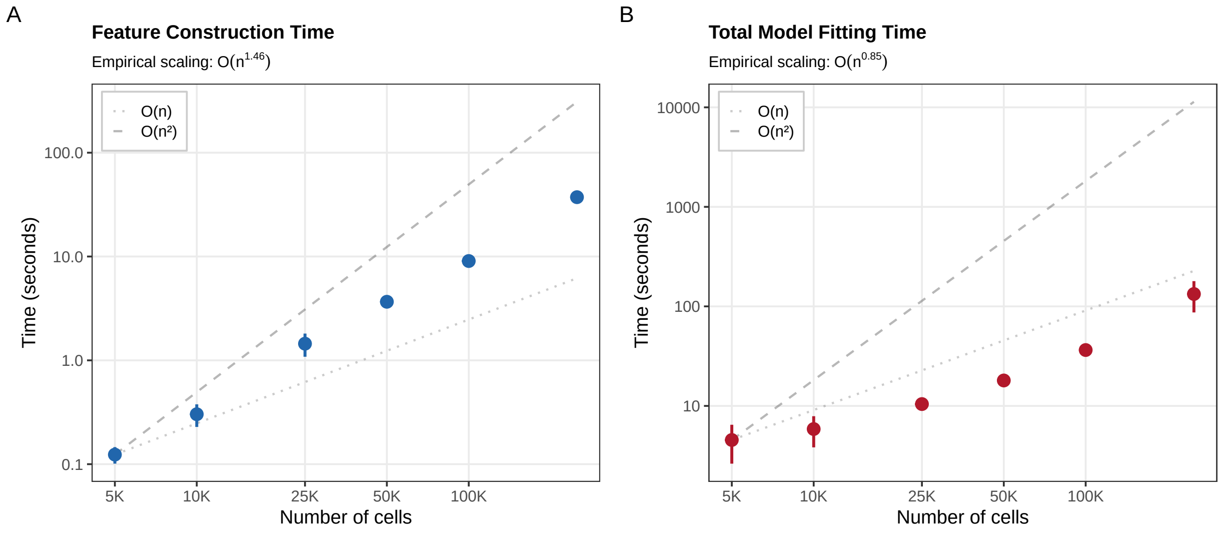

Fig J. Computational scaling of SHADE as a function of cell count. A: Feature construction time shows near-quadratic scaling ($O(n^{1.46})$) with cell count, consistent with the $\mathcal{O}(n_{\text{target}} \times n_{\text{source}})$ complexity of pairwise distance computation. B: Total model fitting time scales sublinearly ($O(n^{0.85})$) due to parallelization and distance matrix reuse. Points show mean timing across 20 replicates per condition; error bars show ± 1 standard deviation. Dashed and dotted reference lines indicate theoretical $O(n^2)$ and $O(n)$ scaling for comparison.

D.9 Coverage and Type I Error Performance

Beyond detection power (Fig 4 in the main text), we evaluated coverage (do 95% credible bands contain the true SIC?) and Type I error control (false positive rates under the null) across all simulation conditions (Fig K in S1 Text–Fig L in S1 Text).

Note on coverage interpretation: Our coverage metric assesses whether hierarchically-shrunk image-level credible bands contain true unshrunk image-level parameters. Hierarchical models intentionally shrink image-level estimates toward patient means to borrow strength across images, producing narrower credible bands centered closer to patient means than true image-specific values. This results in lower-than-nominal coverage (53–100% vs. 95%) but reflects the intended precision-accuracy tradeoff of hierarchical shrinkage, not miscalibration. Coverage is lowest when source density is high (strong signal enables aggressive shrinkage) and highest when source density is low (weak signal produces conservative estimates).

SHADE Hierarchical with 2–3 images per patient maintains well-controlled median Type I error (0.8–7.5%, comparable to envelope tests). With only 1 image per patient, SHADE exhibits extreme behavior (either overly conservative or poorly calibrated), requiring multiple images for stable performance. SHADE Flat shows severe miscalibration (Type I error: 27–29%, coverage: 53–86%) regardless of conditions, demonstrating hierarchical pooling's critical importance.

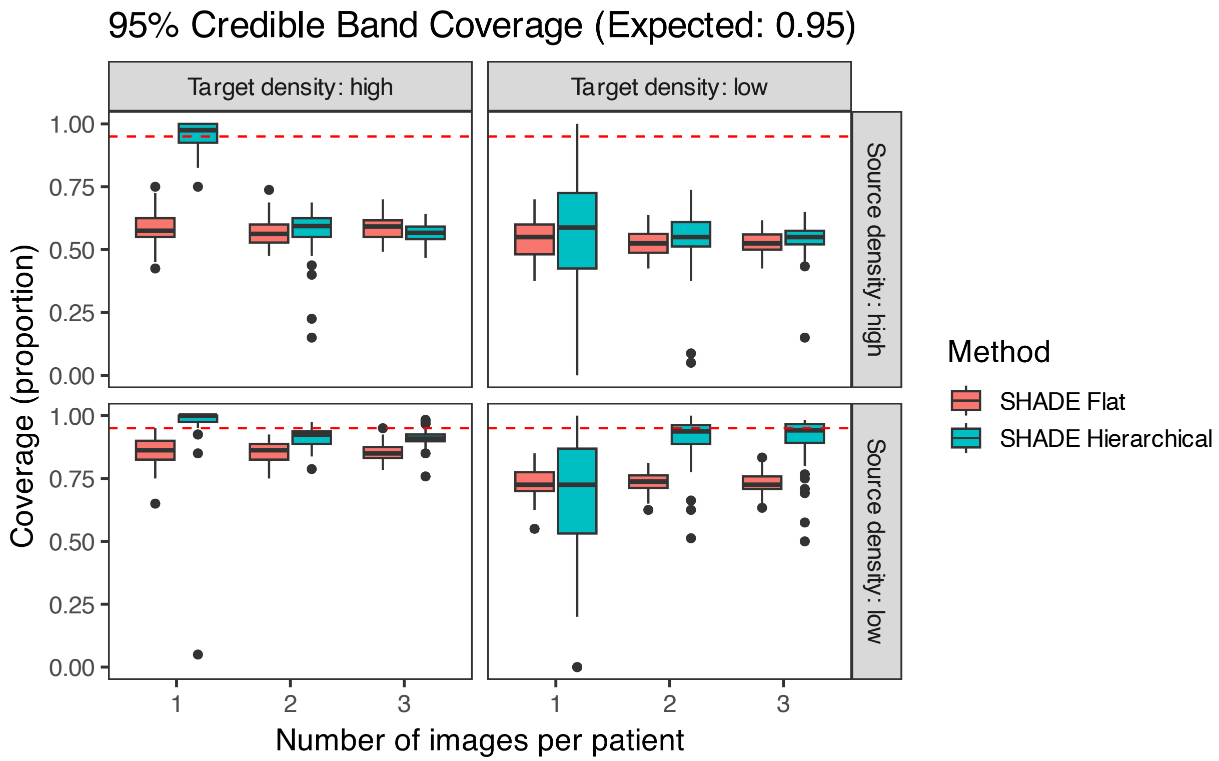

Fig K. Coverage performance of SHADE Hierarchical and SHADE Flat models across simulation conditions. Boxplots show the proportion of images in which 95% simultaneous credible bands fully contain the true SIC across the entire 0–75 μm range, stratified by source cell density (rows: conditioning cell type) and target cell density (columns: modeled cell type), with number of images per patient (1, 2, or 3) shown on the x-axis. The red dashed line indicates the nominal 95% level. Median coverage ranges from 53–100%, with adaptive calibration: highest coverage (up to 100%) when source density is low, lowest coverage (53–59%) when source density is high and target density is low, precisely where detection power is highest. Coverage for SHADE Hierarchical improves substantially with multiple images per patient when both densities are low (1 image: 73%; 2–3 images: 94%).

D.9.1 Practical Recommendations

SHADE Hierarchical requires at least 2 images per patient for stable performance. With multiple images and at least one cell type at moderate-to-high density, SHADE offers superior power with acceptable error control (0.8–7.5%, comparable to envelope tests), making it suitable for exploratory hypothesis generation. For confirmatory studies requiring uniform error control, or when only 1 image per patient is available, envelope tests provide more stable though conservative performance.

D.10 Robustness to Spatial Confounding

We tested SHADE's robustness to unmeasured spatial compartments (e.g., tumor islands, stromal regions) that create baseline density differences independent of cell-cell interactions. We simulated patterns with true

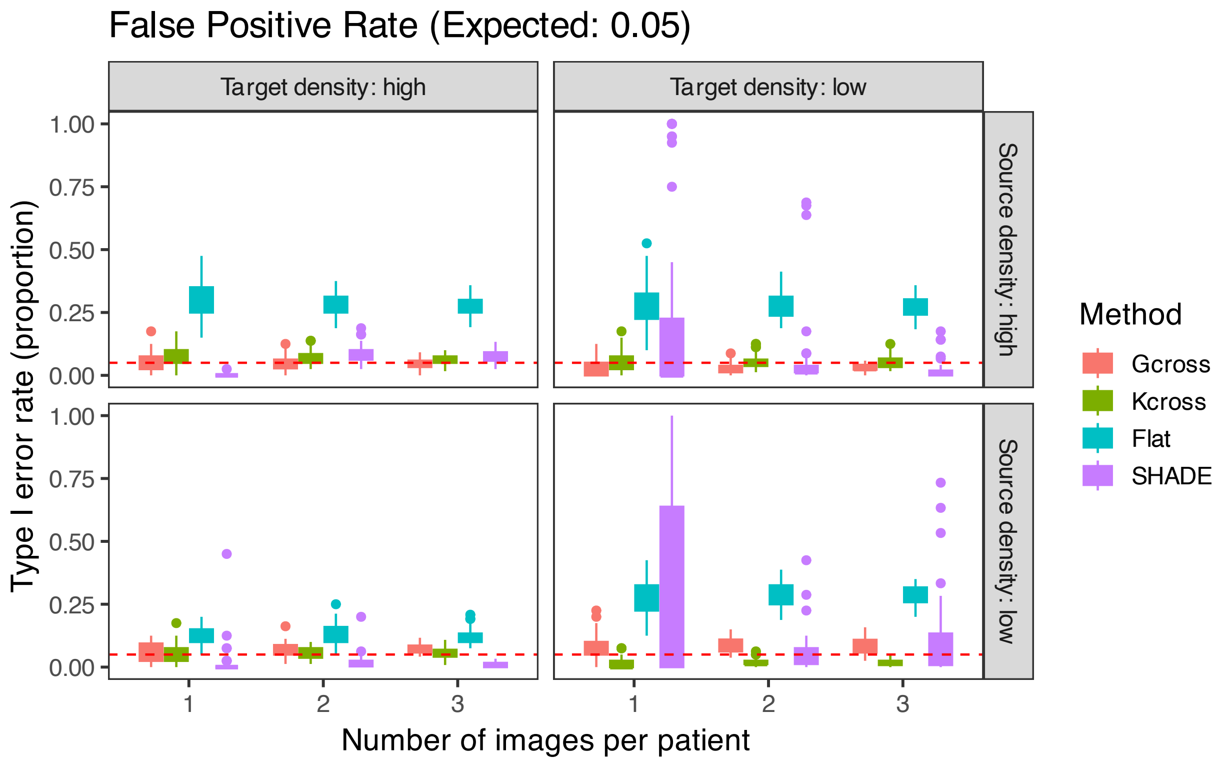

Fig L. Type I error rates across simulation conditions. Boxplots show the proportion of null simulations (true SIC = 0) in which methods incorrectly detect non-zero spatial interactions, for SHADE Hierarchical, SHADE Flat, G-cross, and K-cross methods. Results are stratified by source cell density (rows: conditioning cell type) and target cell density (columns: modeled cell type), with number of images per patient (1, 2, or 3) shown on the x-axis. The red dashed line indicates the nominal 5% level. SHADE Hierarchical with 2–3 images per patient exhibits well-controlled median type I error rates (0.8–7.5%) comparable to envelope tests (G-cross: 2.5–8.8%; K-cross: 1.3–6.7%), though with greater variability. With only 1 image per patient, SHADE Hierarchical shows extreme behavior (0% or high variability). SHADE Flat shows severe inflation (median 27–29%) regardless of number of images.

source-target interactions plus compartment effects (multiplicative baseline intensity: 0.8, 1.2, or 1.5) across 3 spatial regions, then fit SHADE models ignoring compartment structure. All simulations used 3 images per patient and 3 compartments defined by Voronoi tessellation. We assessed detection power, coverage (do 95% credible bands contain the true SIC?), and Type I error (false detection when only compartment effects exist).

D.10.1 Key Findings

SHADE's performance under spatial confounding depends critically on cell densities (Fig M in S1 Text–Fig P in S1 Text). When both source and target densities are high, SHADE achieves perfect power (100%) but severely undercovers (43–52% vs. nominal 95%) and exhibits elevated Type I error (11.7–17.1%) that worsens with compartment strength. The method confidently estimates interactions but conflates true spatial

associations with compartment-induced heterogeneity. When target density is low, wider uncertainty bands provide partial robustness: coverage improves (82–93%) and Type I error drops (1.7–5.8%), though at the cost of reduced power. When both densities are sparse, SHADE becomes appropriately conservative (92–93% coverage, 1.3–2.9% Type I error) but loses detection sensitivity (27–30% power). SHADE Flat consistently exhibits higher false positive rates (26–40%) than SHADE Hierarchical across all regimes, demonstrating hierarchical pooling's protective effect even under model misspecification.

D.10.2 Implications for Practice

These results demonstrate that unmeasured spatial heterogeneity—compartments, regional density differences, or tissue architecture effects—can produce substantial bias in estimated spatial interactions. When such structure is suspected, analysts should: (1) explicitly model compartments if boundaries can be identified (e.g., tumor/stroma annotations), (2) interpret strong positive findings with caution in high-density scenarios where confounding bias is most severe, or (3) conduct sensitivity analyses by comparing SHADE estimates with and without suspected confounders included. The regime-dependent nature of the bias suggests that data characteristics (target/source densities, number of images) interact with model misspecification to determine whether SHADE maintains calibration or produces biased inference.

E Extended results for colorectal cancer analysis

E.1 Detailed description of colorectal cancer dataset and model preparation

The colorectal cancer (CRC) dataset used in this study is a publicly available collection of multiplexed tumor tissue images from 35 patients [Schürch et al., 2020]. Each patient contributed four images, each derived from separate biopsies, yielding a total of 140 images. Images were annotated with single-cell resolution across 16 cell types and 56 protein markers, resulting in a multilevel structure: images nested within patients, patients nested within two immune phenotype groups—Crohn's-like reaction (CLR) and diffuse inflammatory infiltration (DII).

The dataset contains approximately 200,000 cells. For analysis, we focused on the eight most abundant cell types. Cell labels were refined to better reflect marker-based characterization: "stroma" cells were reclassified as hybrid epithelial-mesenchymal (E/M) cells based on co-expression of cytokeratin and vimentin [Kuburich et al., 2024], while "smooth muscle" cells were relabeled as cancer-associated fibroblasts (CAFs) due to expression of α-SMA and vimentin [Cao et al., 2025]. Additional cell types included CD163$^+$ macrophages (TAMs), CD8$^+$ T cells, granulocytes, memory CD4$^+$ T cells, tumor cells, and vasculature.

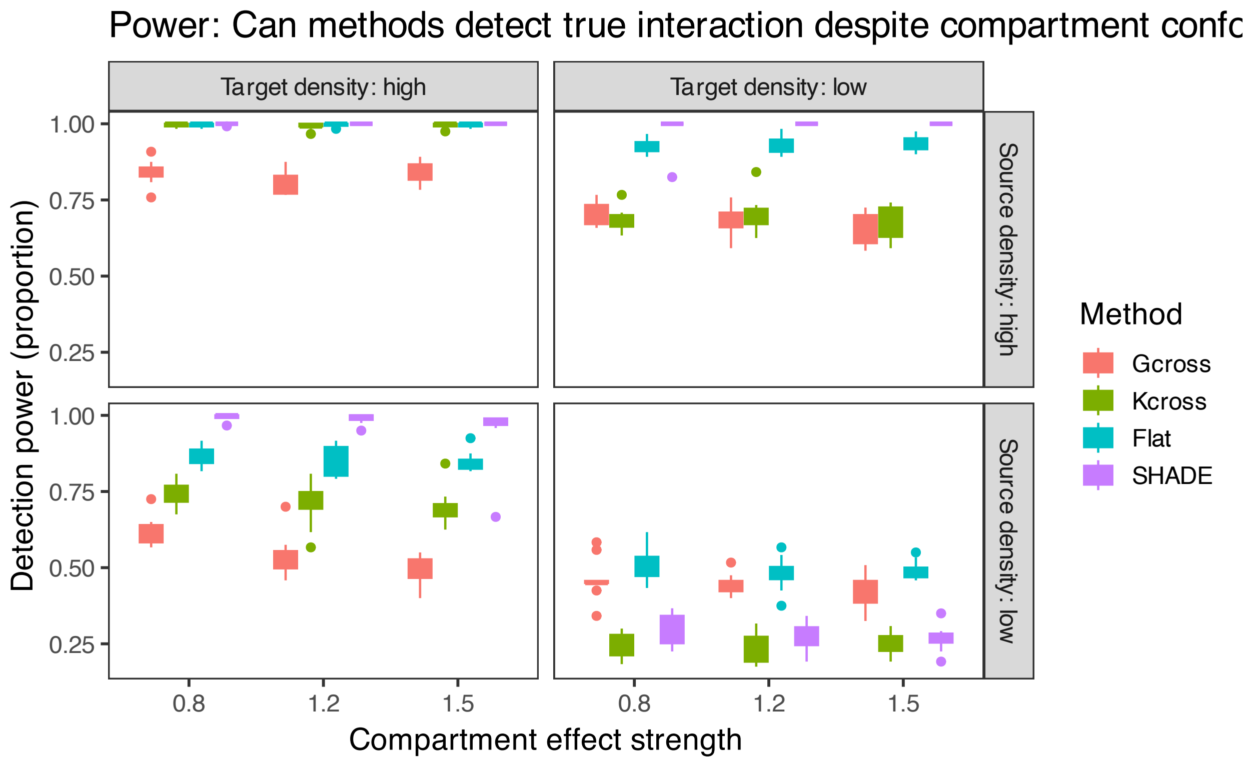

Fig M. Detection power for SHADE and envelope test methods in the presence of compartment confounding. Boxplots show the proportion of datasets in which methods correctly identify non-zero spatial interactions despite the presence of unmeasured compartment structure. Results are stratified by source cell density (rows: conditioning cell type) and target cell density (columns: modeled cell type), with compartment effect strength (0.8, 1.2, 1.5) shown on the x-axis. All simulations used 3 images per patient and 3 spatial compartments. SHADE Hierarchical maintains perfect power (100% median) when either source or target density is high, but power drops to 27–30% when both densities are low.

We selected target populations ($CD8^+$ T cells, memory $CD4^+$ T cells, and granulocytes) based on their functional relevance to anti-tumor immunity, and source populations (vasculature, tumor cells, CAFs, TAMs, hybrid E/M cells) based on their roles in tissue architecture and immune modulation. Vasculature structures infiltration pathways, tumor cells and CAFs contribute to immune exclusion, TAMs modulate local inflammation and immune suppression, and hybrid E/M cells may influence spatial dynamics through motility and stromal interactions.

For each target cell type, we constructed a quadrature scheme by generating 1,000 dummy points per image per cell type. To capture distance-dependent spatial interactions, we constructed interaction features $\boldsymbol{q}_{A_k}(v)$ using a set of three radial basis functions ϕ_p. Data preprocessing included normalization of coordinates and preparation of covariates and interaction features.

Model fitting was performed using variational inference with 1,000 posterior draws after fitting. SICs were estimated jointly for all source cell types with respect to each target, providing interpretable, distance-

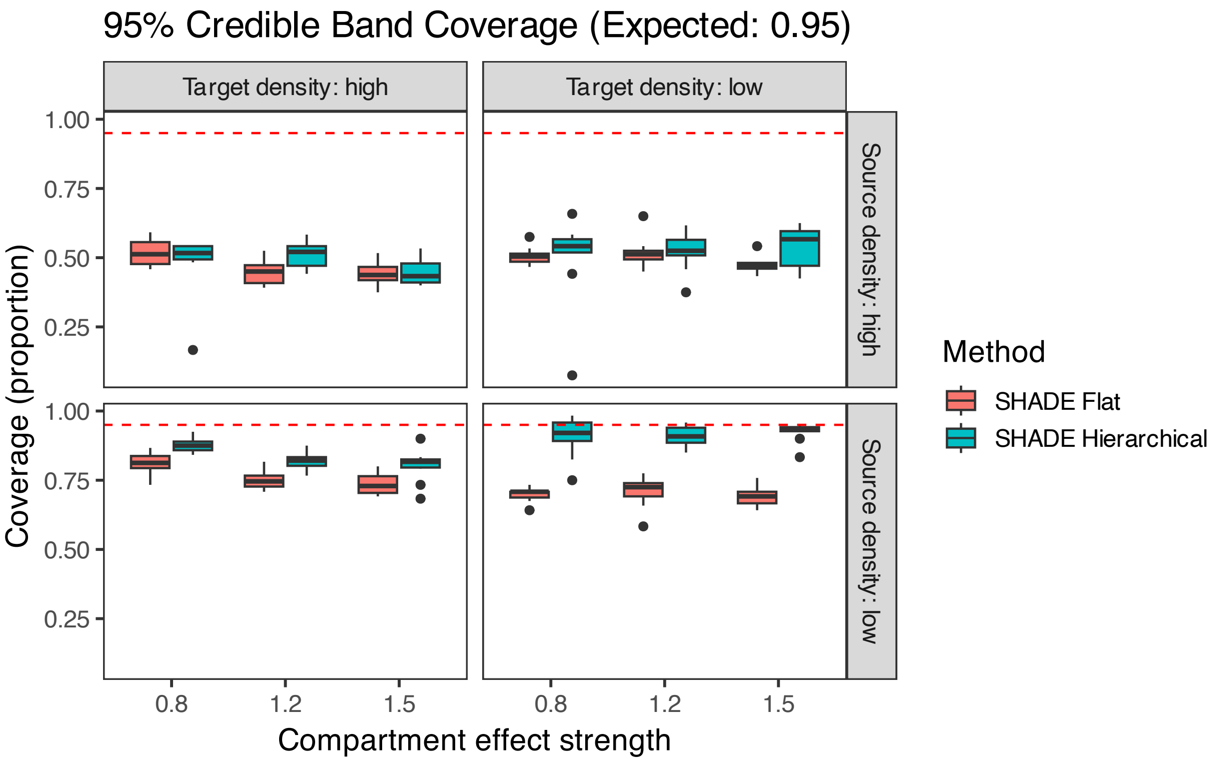

Fig N. Coverage performance in the presence of compartment confounding. Boxplots show the proportion of images in which 95% simultaneous credible bands fully contain the true source-target SIC. Results are stratified by source and target cell densities, with compartment effect strength on the x-axis. The red dashed line indicates the nominal 95% level. When target density is high and source density is high, SHADE severely undercovers (43–52% coverage), incorrectly attributing compartment effects to source-target interactions. When target density is low, coverage improves substantially (82–93%) as wider credible bands encompass confounding bias. When both densities are low, excellent coverage (92–93%) reflects appropriate conservatism.

resolved summaries of spatial associations. We set r_{min} to the dataset's mean cell radius (8–12 μm depending on cohort) and restricted visualization to $r \geq r_{\text{min}}$.

E.2 Compartment-adjusted models

To test robustness to spatial confounding from tissue architecture, we fit compartment-adjusted models incorporating a binary compartment covariate. Compartments were defined based on local tumor cell density: regions with high tumor cell density (> 50th percentile) were classified as tumor-enriched, while remaining regions were classified as stromal. This binary compartment indicator was added as a covariate $z_{\text{comp}}(v) \in \{0, 1\}$ to the conditional intensity function (Equation 2 in main text), with coefficient β_{comp} capturing the effect of compartment on target cell density independent of spatial interactions with source cell types.

Models were fit identically to the primary analysis with the addition of this covariate term. The compart-

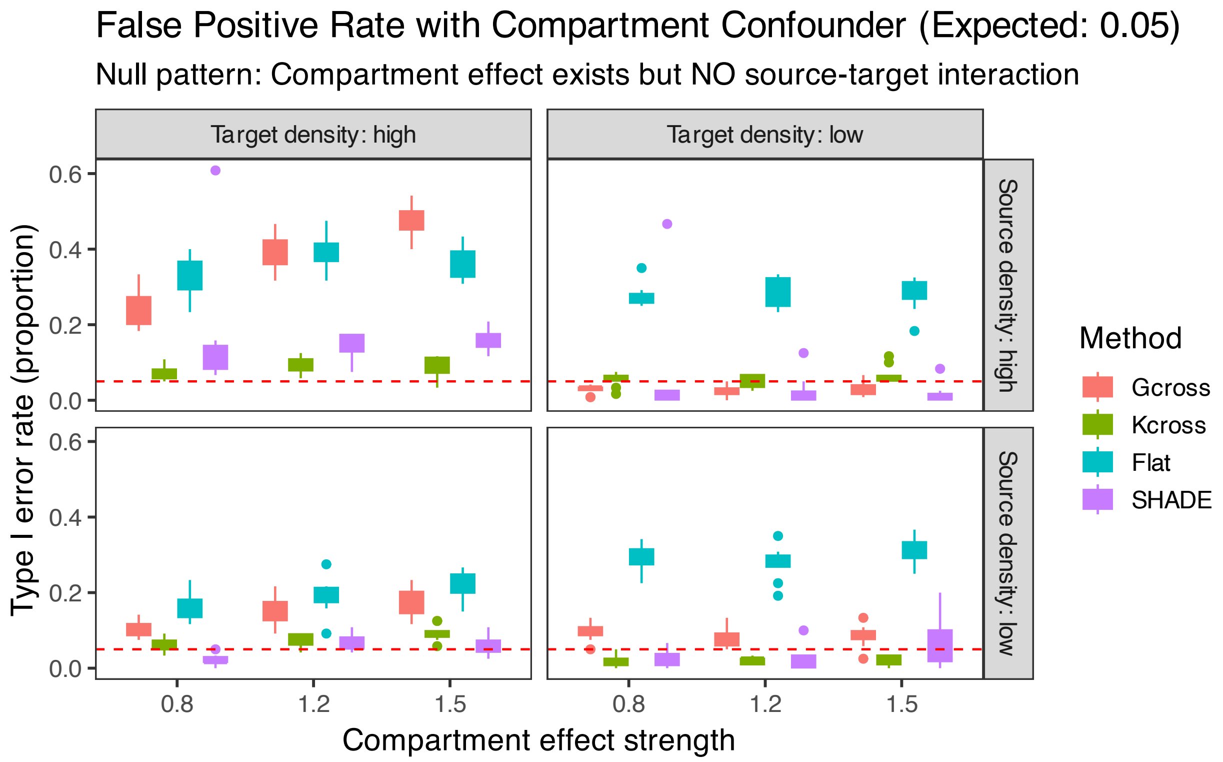

Fig O. Type I error rates when only compartment effects exist (no true source-target interaction). Boxplots show the proportion of null simulations in which methods incorrectly detect non-zero spatial interactions due to unmeasured compartment structure. The red dashed line indicates the nominal 5% level. When both target and source densities are high, SHADE Hierarchical exhibits elevated false positive rates that increase with compartment strength (11.7–17.1%). When target density is low, Type I error is well-controlled (1.7–5.8%). SHADE Flat shows consistently inflated Type I error rates (26–40%) across all conditions, while envelope tests maintain more stable control (G-cross: 2.1–47.1%; K-cross: 1.7–10%).

ment coefficient was estimated separately for CLR and DII patient groups to allow for potential group-specific compartment effects. Table C in S1 Text shows the estimated compartment effects for each target cell type and patient group.

Granulocytes showed significant depletion in tumor-enriched regions, particularly in CLR patients (mean effect = -0.15, 95% CI: -0.22 to -0.08), while CTLs and memory CD4+ T cells showed minimal compartment effects with credible intervals overlapping zero. Despite these compartment effects, spatial interaction patterns remained largely unchanged after adjustment (Fig Q in S1 Text), validating that the null CLR vs DII differences observed in the main analysis are not artifacts of tissue architecture confounding.

E.3 Detailed biological interpretation of CLR vs DII spatial patterns

The cohort-level spatial interaction curves shown in Fig 8 of the main text reveal several notable differences between CLR (immune-infiltrated) and DII (immune-excluded) patient groups. While these differences do

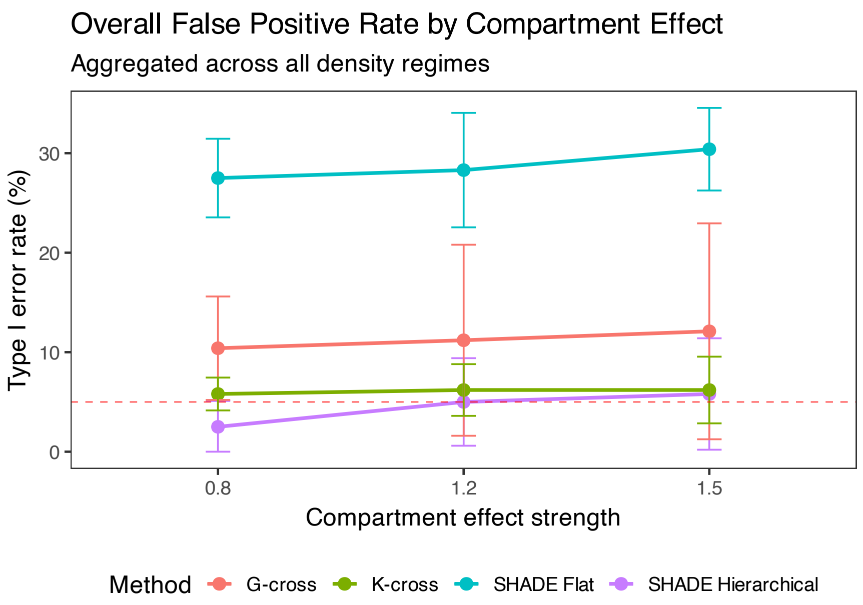

Fig P. Overall Type I error rates by compartment effect strength, aggregated across all density regimes. Line plot shows median Type I error (points) with IQR error bars. All methods exhibit increasing false positive rates as compartment effect strength increases, with SHADE Flat consistently showing the highest rates (26–33%). SHADE Hierarchical maintains the best control (6.2–9.6% median) but still exceeds the nominal 5% level. This demonstrates that unmeasured spatial confounding produces systematic bias that worsens with confounder strength.

not reach statistical significance and require validation in independent cohorts, we provide here a detailed mechanistic interpretation of the observed patterns in the context of known tumor microenvironment biology.

E.3.1 DII-enriched spatial patterns

DII patients exhibited greater clustering of CTLs around tumor cells across all spatial ranges. While this might initially suggest immune recognition, it may alternatively indicate ineffective or exhausted T cell responses that fail to clear tumor cells. Persistent CTL-tumor colocalization without effective cytolysis has been associated with immune dysfunction and tumor immune escape [Dolina et al., 2021, Raskov et al., 2021]. The sustained spatial proximity despite poor tumor control could reflect T cells trapped in a dysfunctional state, unable to eliminate their targets despite close contact.

Similarly, DII patients showed greater attraction of granulocytes to tumor-associated macrophages (TAMs) at all distances, suggesting enhanced myeloid-myeloid interactions that may contribute to immunosuppressive networks. TAMs and granulocytes can form mutually reinforcing suppressive circuits in the tumor microenvironment, with TAMs recruiting and activating immunosuppressive neutrophils, and neutrophils in turn supporting M2-like macrophage polarization. The enhanced spatial co-localization in DII tumors may reflect the establishment of such suppressive myeloid networks that exclude effective adaptive immunity.

Table C. Compartment covariate effects on target cell densities. Values represent posterior mean log-intensity effects (95% credible intervals) of tumor-enriched compartments relative to stromal regions. Negative values indicate depletion in tumor compartments.

Target Cell Type	Group	Mean Effect	95% CI
CTLs (CD8+ T cells)	CLR	0.01	(-0.04, 0.05)
CTLs (CD8+ T cells)	DII	0.00	(-0.04, 0.05)
Memory CD4+ T cells	CLR	0.03	(-0.02, 0.08)
Memory CD4+ T cells	DII	0.02	(-0.04, 0.07)
Granulocytes	CLR	-0.15	(-0.22, -0.08)
Granulocytes	DII	-0.06	(-0.13, 0.02)

E.3.2 CLR-enriched spatial patterns

In contrast, CLR patients demonstrated distinct spatial patterns suggesting different modes of immune-tumor-stroma organization. Hybrid epithelial-mesenchymal (E/M) cells showed stronger attraction of CTLs at short ranges in CLR compared to DII patients. CD4+ T cells have been implicated in modulating epithelial-mesenchymal transition (EMT) processes [Xie et al., 2025, Milosevic and Östman, 2024], and the proximity of CTLs to hybrid E/M cells in CLR tumors may reflect active immune engagement with tumor cells undergoing phenotypic plasticity. Cells in hybrid E/M states may present altered antigen profiles or adhesion molecules that promote CTL infiltration and recognition.

Additionally, CLR patients exhibited greater repulsion of CTLs from cancer-associated fibroblasts (CAFs) at medium-to-long ranges compared to DII, and greater repulsion of memory CD4+ T cells from hybrid E/M cells at these distances. CAFs are known to mediate immune suppression via multiple mechanisms including direct inhibition of T cell function and establishment of physical barriers through extracellular matrix remodeling [Jenkins et al., 2022, Freeman and Mielgo, 2020]. The stronger repulsion in CLR tumors may indicate more effective spatial segregation of immune and suppressive stromal compartments, potentially reflecting physical barriers that constrain the spatial distribution of immune cells while still permitting local infiltration. In contrast, the reduced repulsion in DII could reflect barrier disruption or alternative exclusionary mechanisms that globally constrain immune cell access to tumor regions rather than creating localized segregation patterns.

E.3.3 Caveats and validation requirements

These interpretations remain speculative in the absence of functional validation. The dataset lacks direct measurements of T cell exhaustion, cytolytic activity, CAF activation states, or other functional markers that would support these mechanistic hypotheses. The patterns observed may also reflect technical factors such as unmeasured spatial compartments (tumor islands vs. stroma) that confound apparent cell-cell interactions,

as demonstrated in our compartment robustness simulations (Section D.10 in S1 Text).

Future studies should validate these exploratory findings using: (1) independent colorectal cancer cohorts with multiplexed imaging, (2) functional measurements such as proliferation markers (Ki67), exhaustion markers (PD-1, TIM-3, LAG-3), activation markers (granzyme B, perforin), and CAF phenotyping (FAP, α-SMA, podoplanin), (3) orthogonal clinical outcomes such as survival and treatment response, and (4) explicit modeling of tissue compartment boundaries if available.

E.4 Comparison with traditional G-cross-based spatial clustering analysis

To contextualize our SHADE results, we performed an alternative analysis using the G-cross function [Baddeley et al., 2015], a nonparametric estimator of cross-type clustering commonly used in spatial point pattern analysis. This comparison highlights how SHADE differs from traditional methods in its modeling framework and interpretability of results.

For each tissue section, we computed G-cross estimates at distances from 0 to 80 microns and extracted values at key distances (20, 40, 60 microns) for comparison. To quantify whether differences in clustering could predict patient group (CLR vs DII), we fitted logistic regression models with G-cross as the predictor and used FDR-adjusted p-values to identify significant associations. The resulting estimates are visualized as a heatmap of log-odds ratios (log-ORs), indicating the strength and direction of group differences in clustering at each distance.

The G-cross-based analysis (Fig R in S1 Text) reveals several patterns of cell–cell clustering between CLR and DII patients. For instance, TAM–granulocyte interactions showed strong, statistically significant differences at all examined distances (20–60 microns), with higher clustering in DII tumors. SHADE's directional analysis shows concordant results, with DII patients exhibiting greater attraction of granulocytes to TAMs across all distances, suggesting that this marginal clustering pattern reflects genuine directional spatial association. However, G-cross and SHADE diverge for other interactions. SHADE identified stronger CTL-tumor clustering in DII patients and differential stromal interactions (e.g., CTL repulsion from CAFs being stronger in CLR at medium-long range), patterns that are less evident or show different magnitudes in the G-cross analysis. These discrepancies may reflect SHADE's multivariate adjustment for confounding cell types and hierarchical modeling structure, which can reveal conditional dependencies that differ from marginal pairwise associations captured by G-cross.

E.5 Predictive performance: Spatial interactions and target cell organization

Beyond estimating SICs, SHADE predicts the spatial distribution of each target cell type conditional on the source cells, allowing us to assess how well spatial interactions explain observed patterns. This reveals which cell types are more spatially constrained and how predictability varies across images, patients, and tumor subtypes.

For example, in image 47_B from the dataset, we can produce the following predictions of each target cell type, conditional on the source cell types (Fig S in S1 Text). We can see that the target cell types are quite well predicted, especially CTLs. Likely due to their relative paucity, granulocytes are not quite as well predicted in this image (regions of localization are not as distinct).

We calculated AUCs per cell type and per group (Fig V in S1 Text). All three target cell types were better predicted in CLR patients than in DII patients, though there was a relatively large amount of variability in image-level AUCs.

E.6 Additional Figures

E.7 Multilevel Functional PCA Comparison Methodology

To contextualize SHADE's results within the broader landscape of spatial analysis methods, we compared our findings with multilevel functional principal component analysis (mFPCA) applied to classical spatial summary statistics. This comparison provides insight into how SHADE's conditional modeling framework relates to marginal pairwise analyses commonly used in multiplexed imaging.

E.7.1 mFPCA Approach

We used the `mxfda` R package [Wrobel et al., 2024] to perform multilevel functional PCA on G-cross and L-cross functions. For each of the 15 source-target cell type pairs, we computed spatial summary functions separately for CLR and DII patient groups as follows:

1. **Summary function extraction:** For each tissue section, we computed G-cross and L-cross functions at distances $r \in [0, 75]$ μm with 1 μm increments using the `spatstat` package. G-cross uses Kaplan-Meier edge correction; L-cross uses isotropic edge correction.

2. **Multilevel decomposition:** mFPCA decomposes functional variation across hierarchical levels (images nested within patients) without imposing a parametric model on the underlying point process. We specified proportion of variance explained (pve) = 0.99 to retain principal components explaining 99% of total variability.

3. **Group-level summaries:** For each group (CLR, DII), we extracted the population mean function $\mu(r)$ and uncertainty bands constructed as $\mu(r) \pm \sqrt{\lambda_1}\phi_1(r)$, where λ_1 and $\phi_1(r)$ denote the first eigenvalue and eigenfunction. These bands represent ± 1 SD from the first principal component.

E.7.2 Key Methodological Differences

SHADE and mFPCA address complementary questions: SHADE directly models the underlying point process through the conditional intensity $\lambda(v \mid X_{A_1}, \ldots, X_{A_K})$ (Equation 2), leveraging the point-level likelihood for inference. In contrast, functional data analysis (FDA) approaches applied to spatial statistics (e.g., mFPCA of G-cross or K-cross functions) first compute per-image summary statistics, then analyze the resulting curves in a second stage—a two-stage procedure that does not propagate uncertainty from point pattern estimation into functional inference. While both methods can leverage hierarchical structure, SHADE's generative modeling framework directly integrates the spatial point process likelihood, enabling simultaneous estimation of spatial interactions and variance components across biological scales through partial pooling. SHADE provides a generative probabilistic model estimating conditional effects with multivariate adjustment, while mFPCA performs dimension reduction on marginal pairwise summary statistics without modeling the data-generating process.

E.7.3 Results: Comparison of SHADE with mFPCA

To provide additional context for SHADE's results and explore complementary perspectives on group-level spatial organization, we compared SHADE's group-level SICs with functional data analysis (FDA) of marginal pairwise summary statistics. Specifically, we applied multilevel functional principal component analysis (mFPCA) [Wrobel et al., 2024] separately to CLR and DII patient groups, analyzing G-cross and L-cross functions computed for each tissue section.

Unlike SHADE, which models conditional intensities adjusting for all cell types simultaneously within a generative point process framework, mFPCA decomposes functional variation in observed summary statistics across hierarchical levels (images nested within patients). This approach does not provide a probabilistic model of the underlying point process, but instead characterizes dominant modes of variation in the empirical G-cross and L-cross curves. The resulting group-level mean functions with variability bands (mean $\pm$ 1 SD from the first functional principal component) provide a complementary view of pairwise spatial associations. Note that these bands represent descriptive variation captured by the first PC and do not have coverage properties analogous to confidence or credible intervals.

We computed G-cross and L-cross functions for all 15 source-target pairs at distances from 0 to 75 μm, ran mFPCA separately for each group, and extracted the population mean curves with variability bands.

Full mFPCA results for all pairs are provided in Fig Y in S1 Text and Fig Z in S1 Text for G-cross and L-cross, respectively. Visual comparison of SHADE SICs (Fig 8) with mFPCA group curves reveals both concordance and informative differences.

For instance, SHADE identified greater CTL clustering around tumor cells in DII patients across all distances (Figure 8), while G-cross mFPCA curves show the opposite pattern, with CLR patients exhibiting stronger CTL-tumor associations. This discrepancy likely reflects SHADE's multivariate adjustment: after controlling for other cell types, the conditional CTL-tumor interaction is stronger in DII, whereas marginal pairwise statistics capture different aspects of spatial organization. Similarly, SHADE detected greater granulocyte-TAM clustering in DII patients, but G-cross mFPCA shows minimal group differences for this interaction, suggesting that SHADE's hierarchical modeling and conditional framework reveals patterns that are subtle or absent in marginal analyses. Additionally, SHADE identified stronger CTL repulsion from CAFs at medium-long range in CLR patients, a pattern not evident in marginal G-cross analyses. These discrepancies highlight how multivariate adjustment and hierarchical pooling can reveal conditional dependencies that differ from marginal pairwise associations.

Complete mFPCA results for all 15 cell type pairs are provided in Fig Y in S1 Text (G-cross) and Fig Z in S1 Text (L-cross). Each figure displays group-level mean curves with ± 1 SD bands for CLR (blue) and DII (orange) patients, facilitating visual comparison of marginal spatial associations across patient groups.

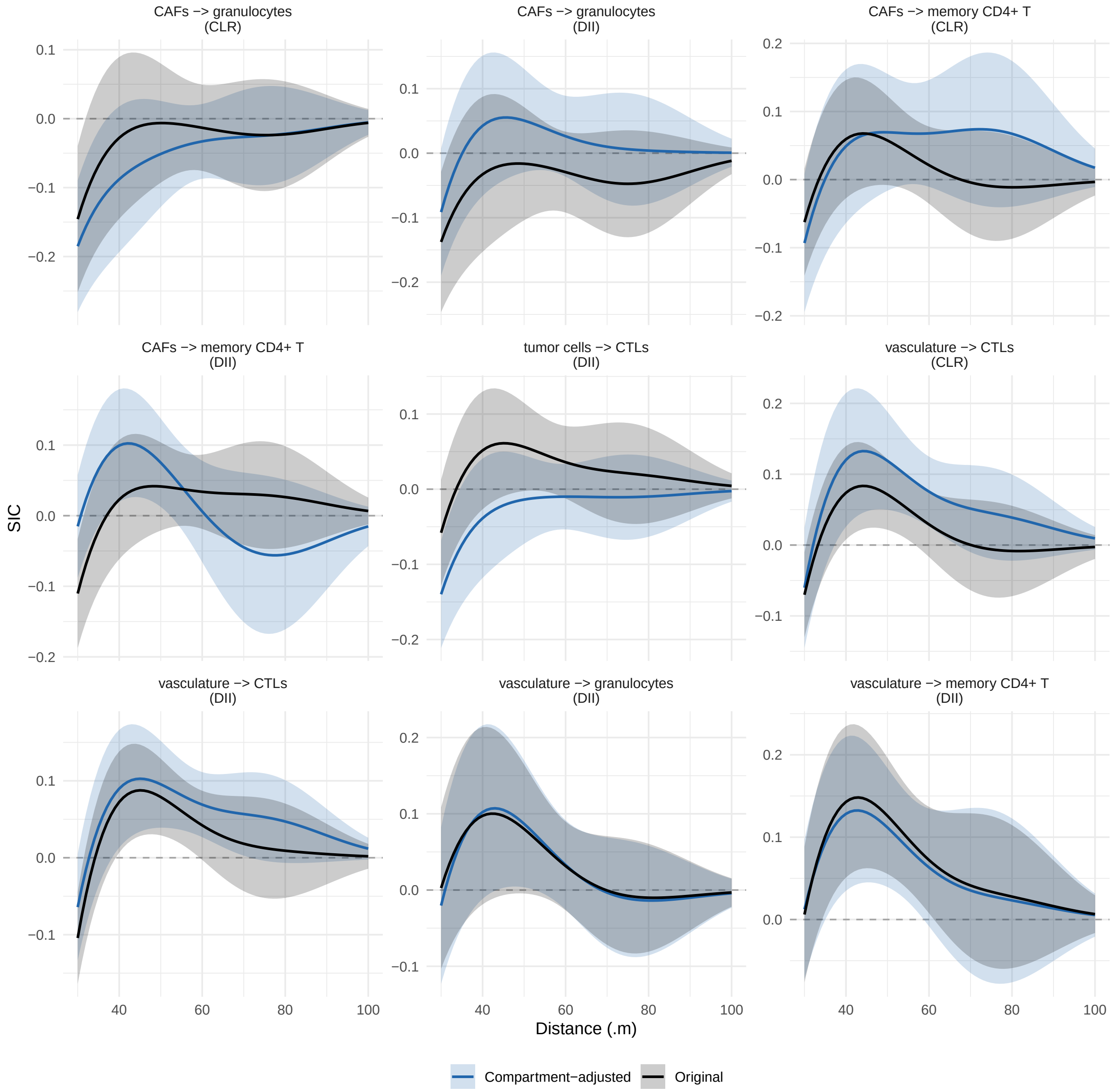

Fig Q. Comparison of original and compartment-adjusted spatial interaction curves for selected source-target pairs showing the largest changes. Spatial interaction patterns remain similar after compartment adjustment, demonstrating that CLR vs DII comparisons are robust to architectural confounding. Shown are the nine interactions with largest absolute changes in SIC estimates; all other pairs show similar concordance.

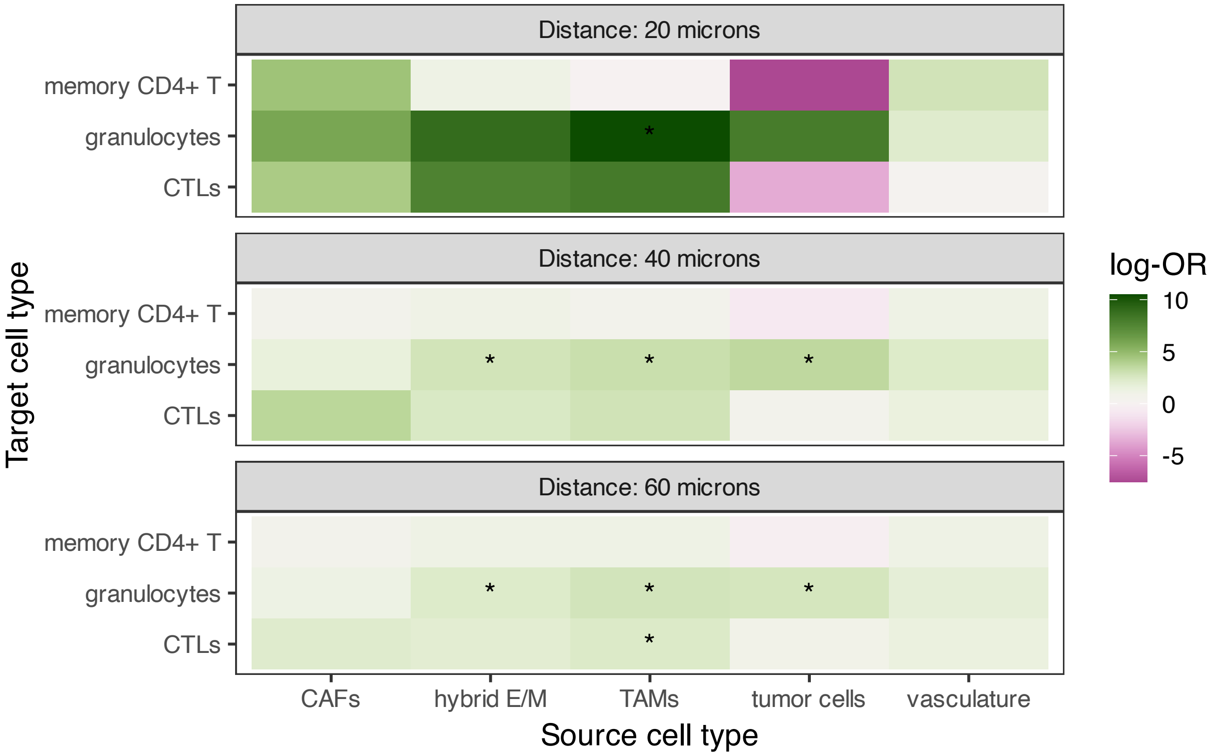

Fig R. Heatmap of log-odds ratios (log-ORs) from logistic regression models comparing G-cross clustering metrics between CLR and DII patient groups, stratified by distance (20, 40, 60 microns). Tiles represent the estimated log-OR for each source–target pair at a given distance, with asterisks indicating significant differences ($p < 0.05$, FDR-adjusted). Green indicates stronger clustering in DII, purple indicates stronger clustering in CLR.

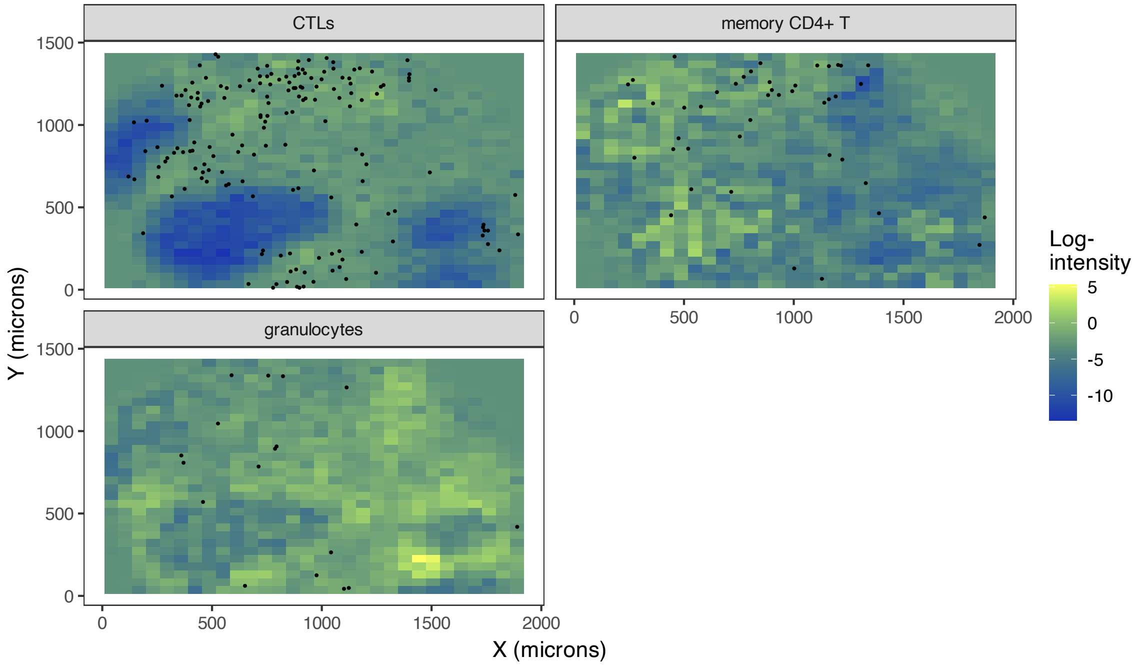

Fig S. Predicted spatial distributions for target cell types in a representative image (47_B), based on the estimated conditional intensity functions. Heat maps show predicted log-intensity; black dots indicate observed cell locations for each target cell type.

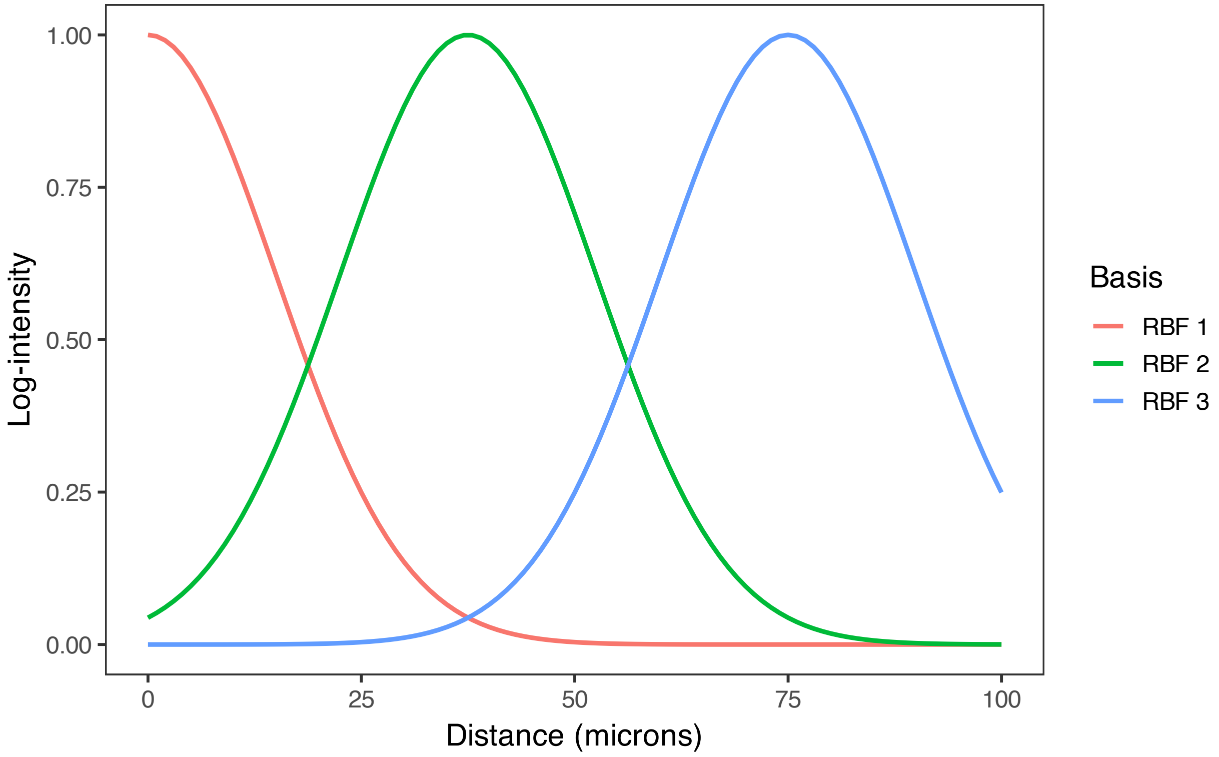

Fig T. Radial basis functions $\phi_p(s)$ used to compute distance-based interaction features $\boldsymbol{q}_{A_k}(v)$ in the CRC analysis. These basis functions define the spatial resolution of the SICs.

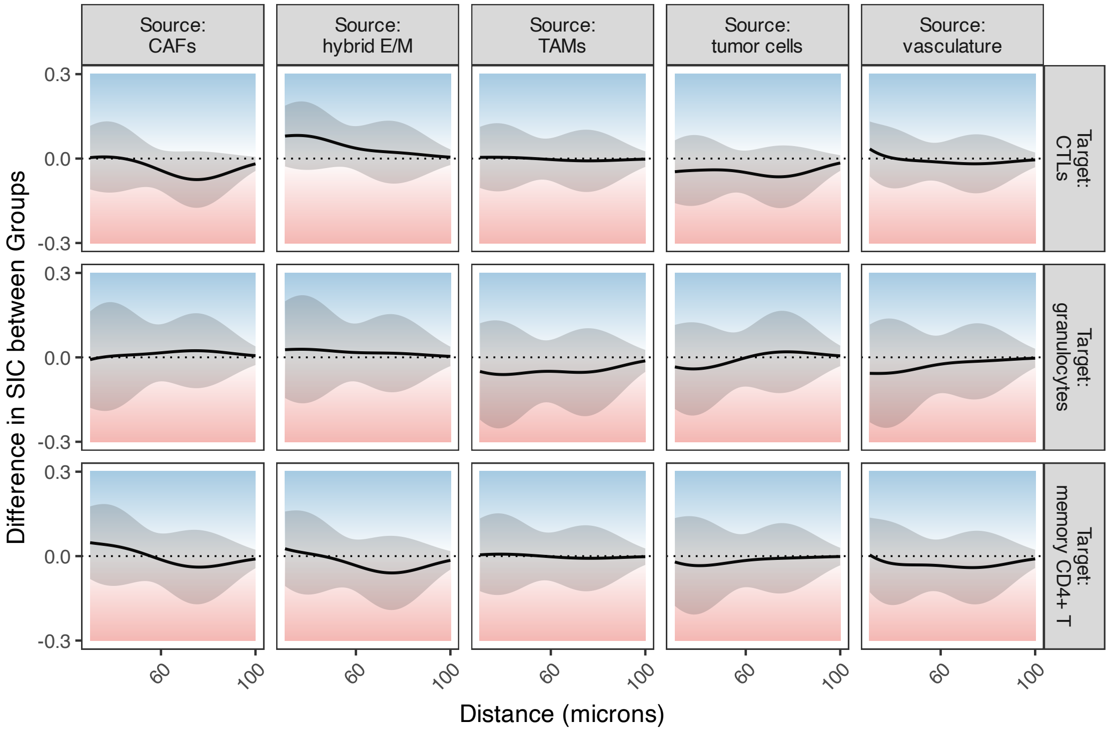

Fig U. The difference in SICs between the two patient groups, with simultaneous 95% credible bands. Curves above 0 (in the blue area) indicate that the log-intensity is greater in CLR patients than DII patients; curves in the red area show the reverse.

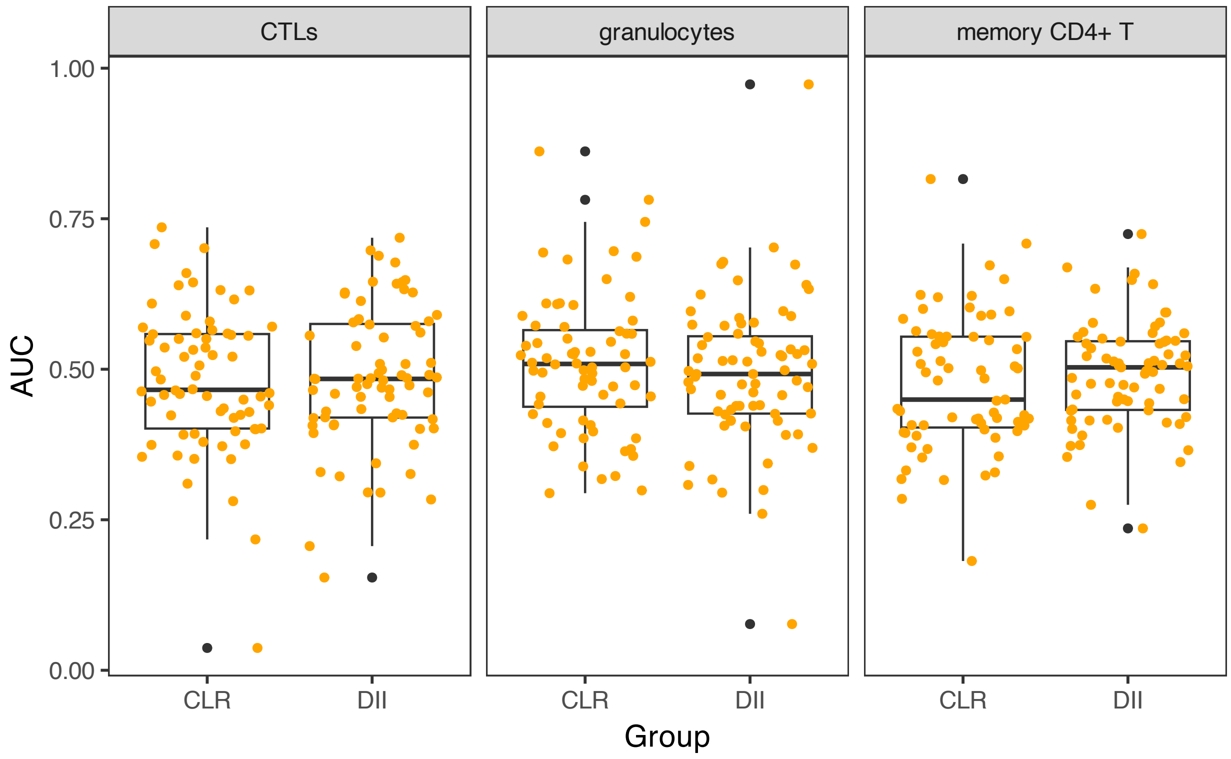

Fig V. Distribution of AUCs by cell type and patient group, evaluating prediction performance of SHADE's conditional intensity model when predicting the spatial organization of each target cell type based on the spatial distribution of all other types.

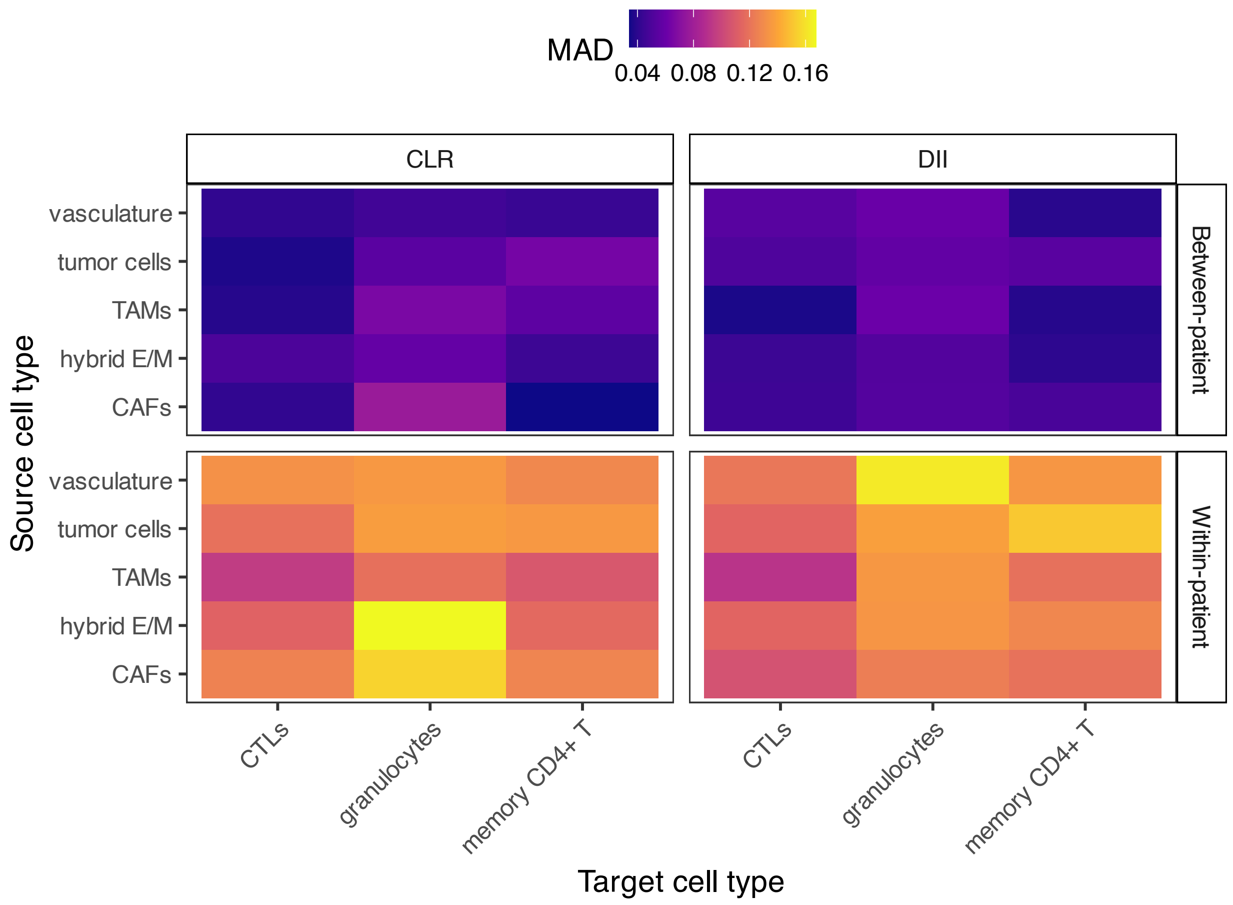

Fig W. Heatmaps show median absolute deviation (MAD) of spatial interaction curve deviations as a measure of heterogeneity for 15 source-target cell type pairs. Top row: between-patient heterogeneity (MAD of patient-level deviations from cohort mean). Bottom row: within-patient heterogeneity (MAD of image-level deviations from patient mean). Left column: CLR tumors. Right column: DII tumors.

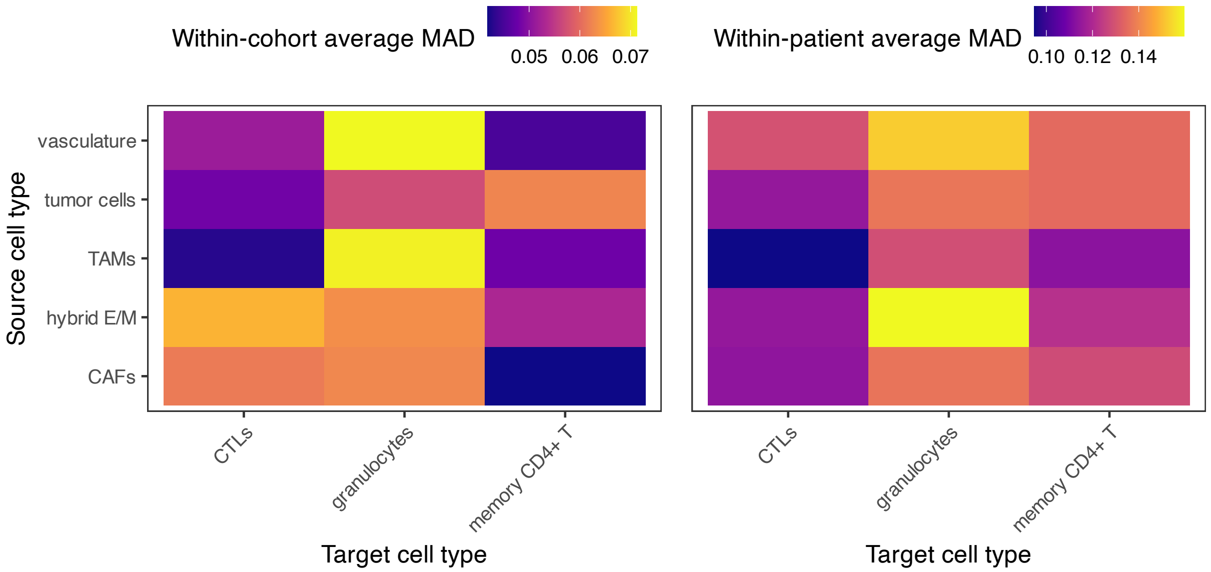

Fig X. Heatmaps show the median absolute deviation (MAD) of SICs across spatial distances, summarizing two sources of variability: between patients (within cohort) and between images (within patient). Higher values indicate greater heterogeneity in spatial structure.

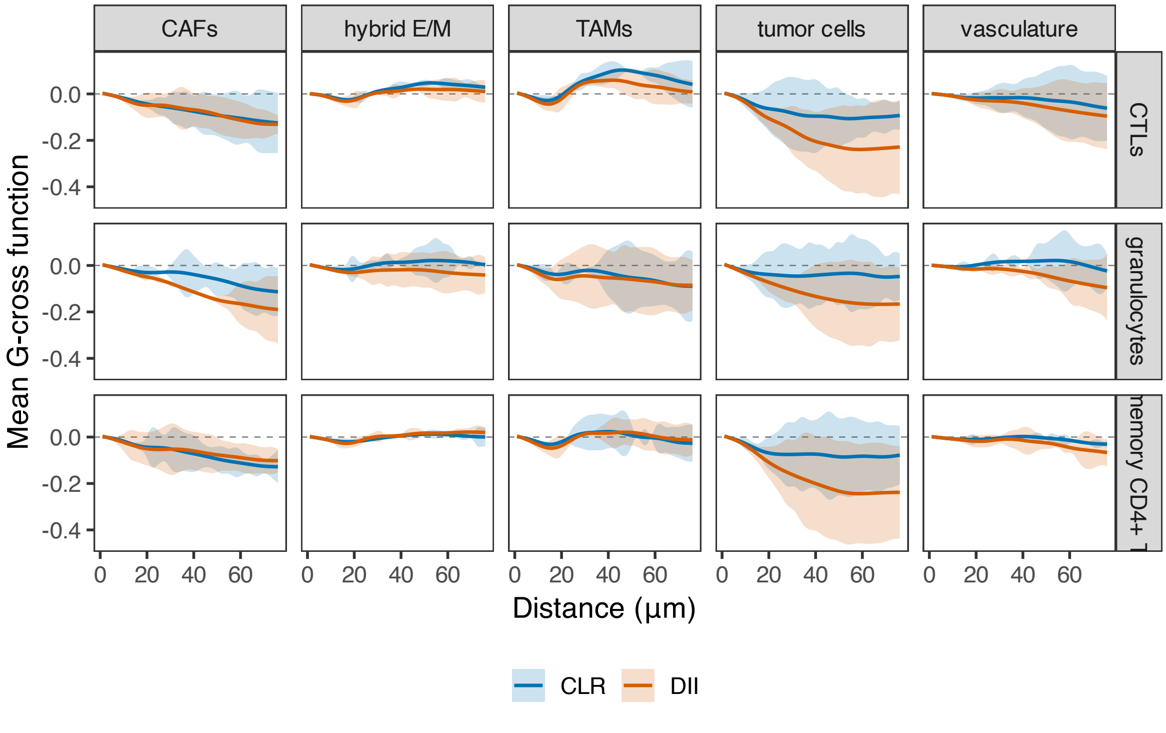

Fig Y. Multilevel functional PCA of G-cross functions for all source-target pairs. For each of 15 cell type pairs, group-level mean G-cross curves are shown for CLR (blue) and DII (orange) patient groups. Ribbons represent ± 1 SD uncertainty bands from the first functional principal component ($\mu(r) \pm \sqrt{\lambda_1}\phi_1(r)$).

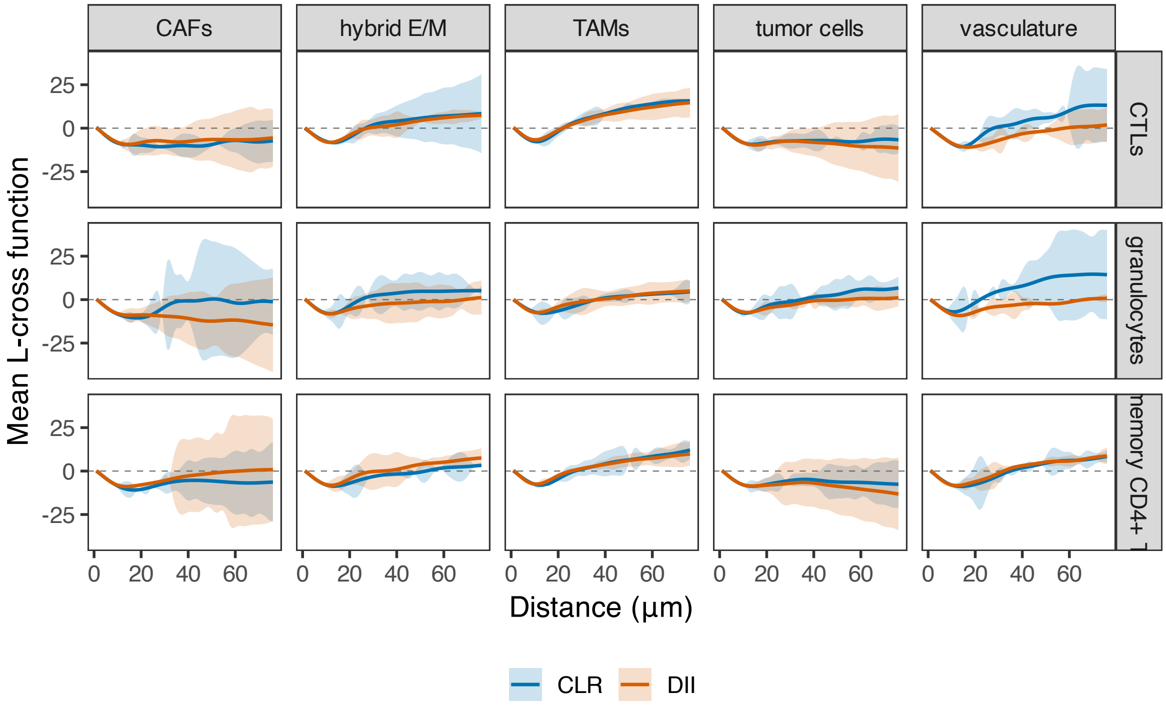

Fig Z. Multilevel functional PCA of L-cross functions for all source-target pairs. For each of 15 cell type pairs, group-level mean L-cross curves are shown for CLR (blue) and DII (orange) patient groups. Ribbons represent ± 1 SD uncertainty bands from the first functional principal component ($\mu(r) \pm \sqrt{\lambda_1}\phi_1(r)$). The L-cross function provides a variance-stabilized alternative to G-cross, facilitating comparison across different spatial scales and cell type combinations.

F Priors for Variance Parameters

We place half-normal priors on the standard deviations of the spatial interaction coefficients at each level of the hierarchy:

$$\sigma_{\text{cohort},p} \sim \text{Half-Normal}(\xi_p, \xi_p), \tag{13}$$

$$\sigma_{\text{patient},p} \sim \text{Half-Normal}(1.5, 10), \tag{14}$$

$$\sigma_{\text{image},p} \sim \text{Half-Normal}(1, 10). \tag{15}$$

where is a user-specified location/scale (in our CRC analysis, we used $\xi_p \in \{5, 3, 1\}$).

G Quantifying Variability in Spatial Interactions

We define SICs hierarchically across cohort, patient, and image levels. For a given source–target cell type pair (A, B), to capture hierarchical variation, we define *patient-level* and *image-level* SICs as:

$$\text{SIC}^{(n)}_{A_k \to B}(s) = \sum_{p=1}^{P} \gamma^{(n,p)}_{A_k} \phi_p(s) \tag{16}$$

$$\text{SIC}^{(m,n(m))}_{A_k \to B}(s) = \sum_{p=1}^{P} \delta^{(m,p)}_{A_k} \phi_p(s) \tag{17}$$

where $\phi_p(s)$ are spline basis functions, and $\gamma^{(n,p)}_{A,B}$ and $\delta^{(m,p)}_{A,B}$ are patient- and image-level coefficients, respectively. To quantify heterogeneity in SICs across patients and images, we compute robust measures of variability at each spatial distance s, and summarize them by taking the median across all distances. Specifically:

Between-patient (within-cohort) variability. Let $\mathcal{C}$ index cohorts, and let $\mathcal{N}_c$ denote the set of patients belonging to cohort $c \in \mathcal{C}$. For each spatial distance s, we compute the cohort-level median SIC as:

$$\overline{\text{SIC}}^{(c)}_{A_k \to B}(s) = \text{median}_{n \in \mathcal{N}_c} \left(\text{SIC}^{(n)}_{A_k \to B}(s) \right)$$

The between-patient (within-cohort) variability for a given cell type pair (A, B) is then defined as:

$$\text{MAD}_{\text{patient}}(A, B) = \text{median}_s \left\{ \text{MAD}_n \left[\text{SIC}^{(n)}_{A_k \to B}(s) - \overline{\text{SIC}}^{(c(n))}_{A_k \to B}(s) \right] \right\} \tag{18}$$

Between-image (within-patient) variability. Similarly, for each patient n, we compute the patient-level median SIC:

$$\overline{\mathrm{SIC}}^{(n)}_{A_k \to B}(s) = \mathrm{median}_{m:n(m)=n} \left(\mathrm{SIC}^{(m,n)}_{A_k \to B}(s) \right)$$

The between-image variability is defined as the MAD of image-level SICs from the patient median:

$$\mathrm{MAD}_{\mathrm{image}}(A, B) = \mathrm{median}_s \left\{ \mathrm{MAD}_m \left[\mathrm{SIC}^{(m,n(m))}_{A_k \to B}(s) - \overline{\mathrm{SIC}}^{(n(m))}_{A_k \to B}(s) \right] \right\} \tag{19}$$

These robust statistics provide interpretable summaries of spatial heterogeneity at each level of the hierarchy while mitigating sensitivity to outliers and small-sample variability. We report these values for each cell type pair and visualize them using heatmaps (Fig X in S1 Text).

References

A. Baddeley, J.-F. Coeurjolly, E. Rubak, and R. Waagepetersen. Logistic regression for spatial Gibbs point processes. *Biometrika*, 101(2):377–392, June 2014. ISSN 0006-3444. doi: 10.1093/biomet/ast060. URL https://doi.org/10.1093/biomet/ast060.

A. Baddeley, E. Rubak, and R. Turner. *Spatial Point Patterns: Methodology and Applications with R*. CRC Press, Nov. 2015. ISBN 978-1-4822-1021-7. Google-Books-ID: rGbmCgAAQBAJ.

M. Berman and T. R. Turner. Approximating Point Process Likelihoods with Glim. *Journal of the Royal Statistical Society Series C: Applied Statistics*, 41(1):31–38, Mar. 1992. ISSN 0035-9254. doi: 10.2307/2347614.

Z. Cao, S. Quazi, S. Arora, L. D. Osellame, I. J. Burvenich, P. W. Janes, and A. M. Scott. Cancer-associated fibroblasts as therapeutic targets for cancer: advances, challenges, and future prospects. *Journal of Biomedical Science*, 32(1):7, Jan. 2025. ISSN 1423-0127. doi: 10.1186/s12929-024-01099-2. URL https://doi.org/10.1186/s12929-024-01099-2.

J. S. Dolina, N. Van Braeckel-Budimir, G. D. Thomas, and S. Salek-Ardakani. CD8+ T Cell Exhaustion in Cancer. *Frontiers in Immunology*, 12:715234, 2021. ISSN 1664-3224. doi: 10.3389/fimmu.2021.715234.

P. Freeman and A. Mielgo. Cancer-Associated Fibroblast Mediated Inhibition of CD8+ Cytotoxic T Cell Accumulation in Tumours: Mechanisms and Therapeutic Opportunities. *Cancers*, 12(9):2687, Sept. 2020. ISSN 2072-6694. doi: 10.3390/cancers12092687. URL https://www.ncbi.nlm.nih.gov/pmc/articles/PMC7564636/.

W. W. Hauck and A. Donner. Wald's Test as Applied to Hypotheses in Logit Analysis. *Journal of the American Statistical Association*, 72(360):851–853, 1977. ISSN 0162-1459. doi: 10.2307/2286473. URL https://www.jstor.org/stable/2286473. Publisher: [American Statistical Association, Taylor & Francis, Ltd.].

H. Högmander and A. Särkkä. Multitype Spatial Point Patterns with Hierarchical Interactions. *Biometrics*, 55(4):1051–1058, 1999. ISSN 1541-0420. doi: 10.1111/j.0006-341X.1999.01051.x. URL https://onlinelibrary.wiley.com/doi/abs/10.1111/j.0006-341X.1999.01051.x. _eprint: https://onlinelibrary.wiley.com/doi/pdf/10.1111/j.0006-341X.1999.01051.x.

L. Jenkins, U. Jungwirth, A. Avgustinova, M. Iravani, A. Mills, S. Haider, J. Harper, and C. M. Isacke. Cancer-Associated Fibroblasts Suppress CD8+ T-cell Infiltration and Confer Resistance to Immune-Checkpoint Blockade. *Cancer Research*, 82(16):2904–2917, Aug. 2022. ISSN 0008-5472. doi: 10.1158/0008-5472.CAN-21-4141. URL https://www.ncbi.nlm.nih.gov/pmc/articles/PMC9379365/.

N. A. Kuburich, J. M. Kiselka, P. den Hollander, A. A. Karam, and S. A. Mani. The Cancer Chimera: Impact of Vimentin and Cytokeratin Co-Expression in Hybrid Epithelial/Mesenchymal Cancer Cells on Tumor Plasticity and Metastasis. *Cancers*, 16(24):4158, Dec. 2024. ISSN 2072-6694. doi: 10.3390/cancers16244158. URL https://www.ncbi.nlm.nih.gov/pmc/articles/PMC11674825/.

V. Milosevic and A. Östman. Interactions between cancer-associated fibroblasts and T-cells: functional crosstalk with targeting and biomarker potential. *Upsala Journal of Medical Sciences*, 129: 10.48101/ujms.v126.10710, May 2024. ISSN 0300-9734. doi: 10.48101/ujms.v129.10710. URL https://www.ncbi.nlm.nih.gov/pmc/articles/PMC11165253/.

J. Moller and R. P. Waagepetersen. *Statistical Inference and Simulation for Spatial Point Processes*. Chapman and Hall/CRC, New York, Sept. 2003. ISBN 978-0-203-49693-0. doi: 10.1201/9780203496930.

M. Myllymäki, T. Mrkvička, P. Grabarnik, H. Seijo, and U. Hahn. Global Envelope Tests for Spatial Processes. *Journal of the Royal Statistical Society Series B: Statistical Methodology*, 79(2):381–404, Mar. 2017. ISSN 1369-7412. doi: 10.1111/rssb.12172.

H. Raskov, A. Orhan, J. P. Christensen, and I. Gögenur. Cytotoxic CD8+ T cells in cancer and cancer immunotherapy. *British Journal of Cancer*, 124(2):359–367, Jan. 2021. ISSN 1532-1827. doi: 10.1038/s41416-020-01048-4. URL https://www.nature.com/articles/s41416-020-01048-4. Number: 2 Publisher: Nature Publishing Group.

D. Ruppert, M. P. Wand, and R. J. Carroll. *Semiparametric Regression*. Cambridge Series in Statistical and Probabilistic Mathematics. Cambridge University Press, Cambridge, 2003. ISBN 978-0-521-78050-6. doi: 10.1017/CBO9780511755453.

C. M. Schürch, S. S. Bhate, G. L. Barlow, D. J. Phillips, L. Noti, I. Zlobec, P. Chu, S. Black, J. Demeter, D. R. McIlwain, S. Kinoshita, N. Samusik, Y. Goltsev, and G. P. Nolan. Coordinated Cellular Neighborhoods Orchestrate Antitumoral Immunity at the Colorectal Cancer Invasive Front. *Cell*, 182(5):1341–1359.e19, Sept. 2020. ISSN 1097-4172. doi: 10.1016/j.cell.2020.07.005.

J. Wrobel, A. C. Soupir, M. T. Hayes, L. C. Peres, T. Vu, A. Leroux, and B. L. Fridley. Mxfda: A comprehensive toolkit for functional data analysis of single-cell spatial data. *Bioinformatics Advances*, 4 (1):vbae155, Jan. 2024. ISSN 2635-0041. doi: 10.1093/bioadv/vbae155.

Y. Xie, X. Wang, W. Wang, N. Pu, and L. Liu. Epithelial-mesenchymal transition orchestrates tumor microenvironment: current perceptions and challenges. *Journal of Translational Medicine*, 23:386, Apr. 2025. ISSN 1479-5876. doi: 10.1186/s12967-025-06422-5. URL `https://www.ncbi.nlm.nih.gov/pmc/articles/PMC11963649/`.
