# Supplementary material for: SHADE: A multilevel Bayesian framework for modeling directional spatial interactions in tissue microenvironments
Source: PLoS Comput Biol. 2026 Feb 4;22(2):e1013930. doi: 10.1371/journal.pcbi.1013930 (PMC12890221; doi:10.1371/journal.pcbi.1013930)
Supplement: Attachment — Submitted filename: Response to Reviewers.pdf [file pcbi.1013930.s002.pdf]

Responses to Reviewers for SHADE

Joel Eliason, Michele Peruzzi, Arvind Rao

December 4, 2025

1 Response to Reviewer 1

We thank the reviewer for their thoughtful comments, which we believe have greatly improved this manuscript. Your feedback helped us clarify key methodological details and expand our comparisons with existing approaches. Below we have provided point-by-point responses to each comment. For easy reference, all manuscript changes are highlighted in blue, in both the responses here as well as the revised manuscript. **The blue text in the reply is a verbatim copy of the changes appearing in the paper**. We greatly appreciate the time and care you brought to reviewing this work!

1. For inference, the method avoids computing the integral term of the Poisson point process likelihood and substitutes an approximate scheme based on logistic regression classification of observed cells versus homogenous Poisson noise. This is motivated at least in part by computational efficiency concerns, yet there doesn't appear to be any discussion of computational efficiency. Spatial technologies are scaling now to hundreds of thousands of cells per sample, so a natural question the authors ought to address is how this method scales. For example, Equation 3 scales quadratically with the number of cells if implemented naively, which would quickly become problematic for many thousands of cells.

   Response: We added a discussion of computational scalability to Section 2.5, including empirical timing experiments demonstrating tractability for large-scale datasets:

   > Feature construction for each focal cell type B involves evaluating inter-cell distances between observed and dummy focal locations and all non-focal cells. This step scales as $\mathcal{O}(n_{\text{focal}} \times n_{\text{source}})$, which is quadratic in the total number of cells when the focal and source sets are of similar size. However, this operation is implemented using the optimized `crossdist` routine from `spatstat.geom`, which efficiently computes pairwise distances in compiled code. The resulting distance matrix is reused across all basis functions $\{\phi_p\}$ and source types $\{A_k\}$, so the dominant cost occurs only once per focal type.
   >
   > To assess the practical runtime implications, we performed a timing experiment varying the total number of cells from 5,000 to 250,000 using variational inference (Supplement, Section S4.8). Feature construction time scaled as

$O(n^{1.46})$ (empirical exponent from log-log regression), while total model fitting time scaled as $O(n^{0.85})$ due to efficient distance matrix reuse. At 100,000 cells, total fitting time was approximately 36 seconds; at 250,000 cells, approximately 133 seconds. These benchmarks demonstrate that SHADE remains computationally tractable for large-scale multiplexed imaging studies.

2. What also frustrates interpretation is that every pair of cells has a negative association at very short distances due to spatial crowding (since cells are not really points, there's a limit to how close their centroids can be). Since most of the spatial associations detected appear to be pretty close range ones, it makes negative associations in particular hard to determine. It's not obvious at what distance we should interpret negative numbers as due to crowding versus due to cellular organization. It wasn't clear to me why there couldn't be a term in the regression to capture this generic crowding effect. Maybe more ambitiously, if full segmentation boundaries are available, cellular distances could be computed as boundary-to-boundary avoiding this issue.

   Response: This is an important point that we appreciate the opportunity to address. Apparent negative associations at very short distances indeed reflect geometric centroid exclusion rather than biologically meaningful repulsion. We now predefine a minimum interaction radius to avoid attributing geometric crowding to biological interactions, as detailed in Section 2.1.1:

   > Because cell centroids cannot occur arbitrarily close in space, very short distances (below approximately 1-2 cell diameters) can reflect geometric crowding, cell-cell contact, or segmentation artifacts, making biological interpretation ambiguous. We therefore predefine a minimum interaction radius $r_{\min} = 25\ \mu$m (approximately 1.5-2 typical cell diameters) and report spatial interaction curves only for $r \geq r_{\min}$. The model is fit using all observed cell locations, but posterior summaries and spatial interaction curves are evaluated and reported only at distances $r \geq r_{\min}$ to focus interpretation on unambiguous intercellular spacing. All band-level posterior probabilities are computed on intervals $I \subseteq [r_{\min}, \infty)$.

   To address this further, we have updated the Discussion to note that future extensions could reduce crowding effects using boundary-to-boundary distances:

   > Future extensions could incorporate functional covariates (marker intensity, proliferation, exhaustion scores) into the SIC framework, enabling joint analysis of spatial structure and functional state, or leverage cell boundary information rather than centroids to better capture contact-based interactions.

3. Some of the positive interaction scores (e.g in Fig. 6 and 8) seem quite small. Since inference is done via sampling, their credible intervals should be available which might tell us what we should consider significant. Fig. 3 does appear to show some type of uncertainty region, though I don't see a description of what exactly it is. More

broadly, I'd like to see some discussion of assessing the overall statistical significance of associations. I would imagine a common use case of SHADE is to test all pairs of annotated cell types in both directions. Should one then inspect plots of all n^2 pairs in both directions, which could be hundreds of pairs, or is there some way to compute an aggregate posterior probability of non-zero effects at some distance?

Response: We thank the reviewer for this important point about uncertainty quantification and screening strategies. We have made three key additions to the manuscript:

Interpreting small effects: We now explain in Section 2.1.1 how SIC values on the log scale correspond to multiplicative changes in cell density:

> This curve represents the expected contribution of type A_k cells to the log-intensity of type B cells as a function of distance s from a type A_k cell. Here, log-intensity refers to the logarithm of the conditional intensity function $\lambda(v)$ (Equation 2), which describes the expected spatial density of type B cells (in cells per unit area) at any location v. Because the model is log-linear, SIC values quantify additive changes on the log-intensity scale, which correspond to multiplicative changes in actual cell density. For example, SIC = 0.2 at distance s implies an $e^{0.2} \approx 1.22\times$ (or 22%) increase in the expected local density of type B cells at radius s from a type A_k cell, while SIC = -0.3 corresponds to an $e^{-0.3} \approx 0.74\times$ decrease (26% reduction).

Simultaneous credible bands and screening strategies: We added a new subsection (Section 2.3) describing uncertainty quantification and prioritization approaches for exploratory analyses with many cell type pairs:

> To quantify uncertainty in estimated SICs and assess statistical significance, we employ simultaneous 95% credible bands that account for multiple comparisons across the distance domain. Unlike pointwise credible intervals, simultaneous bands provide joint coverage across all distances within a specified range, offering stronger protection against false discoveries. Statistical significance can be assessed by examining whether the simultaneous band excludes zero over a distance range of interest. Full implementation details are provided in the Supplement (Section S4.1).
>
> In exploratory analyses involving many cell type pairs ($K(K-1)$ directed pairs for K cell types), we propose summary measures to facilitate prioritization: peak location and magnitude (identifying where the strongest interaction occurs), persistence over biologically relevant distance ranges (quantifying consistent associations within pre-specified intervals), and overall strength (integrating absolute effect sizes over significant regions). These measures can be computed across all source–target pairs and visualized as heatmaps for systematic comparison. Formal definitions are provided in the Supplement (Section S4.2).

All SIC figures now display simultaneous credible bands. Statistical significance is assessed by checking whether bands exclude zero over a distance range of interest.

Minor issues:

1. Some acronyms are used without being defined (e.g. CTLs, TAMs). These are common immunological terms but still should be defined to avoid ambiguity.

   Response: Thank you for catching this oversight. We have now defined all acronyms upon first use in the manuscript. CTLs (cytotoxic T lymphocytes, $CD8^+$ T cells) and TAMs (tumor-associated macrophages, $CD163^+$) are now explicitly defined in the Results section.

2. Figure 10 caption needs more explanation. I'm guessing the black dots are observed cells of the labeled type, but it's not clear.

   Response: Thank you for pointing this out. We have clarified the caption for Figure S19:

   > Heat maps show predicted log-intensity; black dots indicate observed cell locations for each target cell type.

2 Response to Reviewer 2

We thank the reviewer for their thoughtful comments, which we believe have greatly improved this manuscript. Your feedback helped us clarify key methodological details and expand our comparisons with existing approaches. Below we have provided point-by-point responses to each comment. For easy reference, all manuscript changes are highlighted in blue, in both the responses here as well as the revised manuscript. **The blue text in the reply is a verbatim copy of the changes appearing in the paper**. We greatly appreciate the time and care you brought to reviewing this work!

1. Introduction: "... yet existing spatial models typically analyze images independently, potentially overlooking heterogeneity across patients", and similarly abstract: "Many analysis methods ... treat images independently".

   Generally, I feel such statements should be made explicit and substantiated with references. Also, I do not quite understand the point, why would analyzing images independently automatically mean that patient heterogeneity is not studied? And is Shade not actually based on computing statistics (SIC curves) on individual images? In the current form of the ms, I do not find any follow-up on this point in the results or discussion sections, please clarify.

   Response: Thank you for this thoughtful observation. We agree that our original statement was overstated, and we appreciate the opportunity to clarify this important distinction. The Introduction now clarifies the distinction: independent analyses can descriptively characterize heterogeneity by comparing per-image summaries, but cannot formally decompose variance components or perform partial pooling within a unified framework. The revised Introduction now provides explicit references and explains this nuance:

   > Standard pipelines for analyzing spatial cellular interactions in multiplexed imaging data typically compute second-order summary statistics, such as Ripley's K- and L-functions or the G-cross function, independently for each image to quantify tumor–immune interactions, infiltration patterns, or neighborhood structure [Barua et al., 2018, Vu et al., 2022, Canete et al., 2022, Samorodnitsky et al., 2024, Seal et al., 2024, Jing et al., 2025, Janeiro et al., 2024, Soupir et al., 2025]. These per-image summaries are then compared across groups or used as covariates in downstream analyses. Although effective for descriptive comparisons, this approach treats each image as an isolated point pattern and cannot share information across biological replicates during estimation. Multilevel models for replicated point patterns have been proposed [Bagchi and Illian, 2015, Lee et al., 2017, Myllymäki et al., 2014, Bell and Grunwald, 2004, Illian and Hendrichsen, 2010, Wrobel et al., 2024], but they typically model derived summaries via two-stage estimation or use parametric models fit via pseudolikelihood with random effects, rather than jointly estimating flexible interaction functions through full Bayesian multilevel inference at the point-process level.

The reviewer correctly notes that SHADE does compute image-level SICs. Results Section 4.3 now clarifies that these are estimated within a joint hierarchical framework:

> Unlike descriptive spatial statistics that compute separate summaries for each image, SHADE's hierarchical Bayesian model jointly estimates SICs at the image, patient, and cohort levels through partial pooling. This enables formal decomposition and quantification of variability at each biological scale, allowing us to assess both intra- and inter-patient variability and distinguish conserved from patient-specific spatial patterns.

Simulation evidence in Supplement Section S4.4 and 3.1 now demonstrates the benefits of this hierarchical approach:

> We found that on average, and across all spatial scales, the average RMSE of $\delta^{(m,p)}_{t_1 \to t_2}$ coefficients was lower for those estimated by the full hierarchical model than the model that fit images individually, most significantly so for closer-range coefficients (Table S2).

> First, when source density is high (regardless of target density), SHADE achieves excellent median power (100%) across all conditions, substantially outperforming envelope tests when target density is low (SHADE 100% vs. G-cross 77%, K-cross 73%). Abundant source cells provide strong conditioning information that hierarchical pooling can effectively leverage. Conversely, when source density is low, performance depends on whether multiple images are available: with 2–3 images per patient, median power reaches 100%; with only 1 image, power drops to 31% as limited conditioning information per image prevents effective pooling.

The Discussion now further contextualizes SHADE's hierarchical approach relative to traditional spatial statistics:

> A key advantage of SHADE over traditional spatial summary statistics is its ability to formally model heterogeneity across biological scales through hierarchical partial pooling, enabling variance decomposition and more nuanced biological inference than post-hoc image-by-image comparisons.

2. Methods (p. 8), "The SIC summarizes ... This curve represents the expected contribution of type A_k cells to the log-intensity...".

   The term "log-intensity" appears for the first time at this point, and lacks a direct interpretation. What does log-intensity of a cell type physically mean, is it related to the spatial cell density? Or would it be possible to normalize it to the average cell-density in the image, or a set of images? This is critical for interpretation of the SIC curves where log-intensity appears on the y-axis throughout the paper.

   Response: We have added a detailed explanation of log-intensity in Section 2.1.1.

> This curve represents the expected contribution of type A_k cells to the log-intensity of type B cells as a function of distance s from a type A_k cell. Here, log-intensity refers to the logarithm of the conditional intensity function $\lambda(v)$ (Equation 2), which describes the expected spatial density of type B cells (in cells per unit area) at any location v. Because the model is log-linear, SIC values quantify additive changes on the log-intensity scale, which correspond to multiplicative changes in actual cell density. For example, SIC = 0.2 at distance s implies an $e^{0.2} \approx 1.22\times$ (or 22%) increase in the expected local density of type B cells at radius s from a type A_k cell, while SIC = -0.3 corresponds to an $e^{-0.3} \approx 0.74\times$ decrease (26% reduction).

Additionally, we have added further clarification on the normalization term in Section 2.1:

> Finally, the intercept term (β_0) captures baseline log-intensity, effectively normalizing to average target cell density. SIC values then quantify deviations from this baseline as a function of proximity to source cells, ensuring curves are directly comparable across images and patients despite differences in overall cell abundance.

3. Methods (p. 9), "A strong A-¿B SIC indicates that the presence of A is statistically predictive ..." (and similar statements in the following).

   It is not clear to me what statistically predictive means here. Is it possible to construct a statistical test for SIC curves different from spatial randomness, or for SIC curves differing between different image sets? The next section about "Multilevel Bayesian Model" suggests that the authors aim for statistical analysis, but I have trouble finding any clear statistical statements, also in the following chapters where synthetic data and image data sets are analyzed. Figures 4 and 5 show confidence intervals but statements on statistical significance are missing, I suggest evaluating significance using bootstrapping procedures. In Figure 4, it seems that all sown curves are within the confidence of the null (CSR) model, does that mean that the generated synthetic data cannot be distinguished from CSR?

   Response: Thank you for highlighting the need for clearer statistical statements. We have made statistical inference much more explicit throughout the manuscript.

   Bayesian inference and uncertainty quantification: We added Section 2.3 describing simultaneous 95% credible bands that control family-wise error across distances:

   > To quantify uncertainty in estimated SICs and assess statistical significance, we employ simultaneous 95% credible bands that account for multiple comparisons across the distance domain. Unlike pointwise credible intervals, simultaneous bands provide joint coverage across all distances within a specified range, offering stronger protection against false discoveries. Statistical significance can be assessed by examining whether the simultaneous band

> excludes zero over a distance range of interest. Full implementation details are provided in the Supplement (Section S4.1).
>
> In exploratory analyses involving many cell type pairs ($K(K-1)$ directed pairs for K cell types), we propose summary measures to facilitate prioritization: peak location and magnitude (identifying where the strongest interaction occurs), persistence over biologically relevant distance ranges (quantifying consistent associations within pre-specified intervals), and overall strength (integrating absolute effect sizes over significant regions). These measures can be computed across all source–target pairs and visualized as heatmaps for systematic comparison. Formal definitions are provided in the Supplement (Section S4.2).

Clarifying "statistically predictive": We updated Section 2.1.2 explaining what predictive associations mean:

> Our modeling framework captures *predictive* spatial associations, not biological causation. A strong $A \to B$ SIC indicates that type A cell locations are statistically predictive of local type B density, formally meaning that the conditional intensity $\lambda(v \mid X_A)$ differs from the marginal intensity $\lambda_B(v)$. While SICs quantify the strength and direction of spatial predictability, they do not establish causal biological effects. The model can incorporate spatial covariates (e.g., distance to tumor margin) to adjust for confounding from first-order intensity effects.

Testing and comparison: As mentioned previously, to test departure from spatial randomness, we now check if simultaneous bands exclude zero. To compare patient groups, we can also compute e.g. difference curves with simultaneous bands e.g. (Figure S21); if bands exclude zero over a distance range, groups differ significantly. All SIC figures now display these bands, and Results sections explicitly state when bands exclude zero.

Figure 4 and CSR: Figure 3 shows one simulation example: while G-cross and L-cross envelopes sometimes contain the observed curves, SHADE's credible bands correctly exclude zero where the ground truth exhibits non-zero interactions, demonstrating that the data can be distinguished from CSR. Panel (d) also shows that SHADE Hierarchical (red) estimates the ground truth more accurately than SHADE Flat (blue), illustrating the benefit of hierarchical pooling. Across all replicates, SHADE achieves high detection rates (Figure 4).

4. In the same context, it would make sense to include more methods for comparison (in addition to G-cross), for example Ripley's K-function is mentioned in the introduction.

   Response: We have now added Ripley's cross-type K-function (K_{AB}, specifically the L-cross transformation) to both the simulation comparison and colorectal cancer analysis.

   In the simulation study (Section 3.1), we added a comparison of SHADE against K-cross envelope tests:

> For comparison, we evaluated three baseline approaches: (1) G-cross envelope tests [Baddeley et al., 2015], testing whether the observed nearest-neighbor distribution falls outside 95% global envelopes constructed from 99 completely spatially random (CSR) simulations; (2) K-cross (specifically, L-cross) envelope tests, using cumulative counts rather than nearest-neighbor distances; and (3) a 'Flat' model that estimates SICs independently for each image without hierarchical pooling. Type I error rates were assessed using null simulations with zero spatial interactions.

Results (Figure 4) now show SHADE outperforms both G-cross and K-cross in detecting associations under low cell densities and limited sampling.

To address this, Section S5.4 now presents the comparison:

> The G-cross-based analysis (Figure S18) reveals several patterns of cell–cell clustering between CLR and DII patients. For instance, TAM–granulocyte interactions showed strong, statistically significant differences at all examined distances (20–60 microns), with higher clustering in DII tumors. SHADE's directional analysis shows concordant results, with DII patients exhibiting greater attraction of granulocytes to TAMs across all distances, suggesting that this marginal clustering pattern reflects genuine directional spatial association. However, G-cross and SHADE diverge for other interactions. SHADE identified stronger CTL-tumor clustering in DII patients and differential stromal interactions (e.g., CTL repulsion from CAFs being stronger in CLR at medium-long range), patterns that are less evident or show different magnitudes in the G-cross analysis. These discrepancies may reflect SHADE's multivariate adjustment for confounding cell types and hierarchical modeling structure, which can reveal conditional dependencies that differ from marginal pairwise associations captured by G-cross.

We have also added Section S5.7.3 comparing SHADE with mFPCA analysis of G-cross and L-cross functions:

> To provide additional context for SHADE's results and explore complementary perspectives on group-level spatial organization, we compared SHADE's group-level SICs with functional data analysis (FDA) of marginal pairwise summary statistics. Specifically, we applied multilevel functional principal component analysis (mFPCA) [Wrobel et al., 2024] separately to CLR and DII patient groups, analyzing G-cross and L-cross functions computed for each tissue section.
>
> Unlike SHADE, which models conditional intensities adjusting for all cell types simultaneously within a generative point process framework, mFPCA decomposes functional variation in observed summary statistics across hierarchical levels (images nested within patients). This approach does not provide a probabilistic model of the underlying point process, but instead

> characterizes dominant modes of variation in the empirical G-cross and L-cross curves. The resulting group-level mean functions with variability bands (mean $\pm$ 1 SD from the first functional principal component) provide a complementary view of pairwise spatial associations. Note that these bands represent descriptive variation captured by the first PC and do not have coverage properties analogous to confidence or credible intervals.

To address this, Section S5.7.3 now presents:

> For instance, SHADE identified greater CTL clustering around tumor cells in DII patients across all distances (Figure 8), while G-cross mFPCA curves show the opposite pattern, with CLR patients exhibiting stronger CTL-tumor associations. This discrepancy likely reflects SHADE's multivariate adjustment: after controlling for other cell types, the conditional CTL-tumor interaction is stronger in DII, whereas marginal pairwise statistics capture different aspects of spatial organization. Similarly, SHADE detected greater granulocyte-TAM clustering in DII patients, but G-cross mFPCA shows minimal group differences for this interaction, suggesting that SHADE's hierarchical modeling and conditional framework reveals patterns that are subtle or absent in marginal analyses. Additionally, SHADE identified stronger CTL repulsion from CAFs at medium-long range in CLR patients, a pattern not evident in marginal G-cross analyses. These discrepancies highlight how multivariate adjustment and hierarchical pooling can reveal conditional dependencies that differ from marginal pairwise associations.

5. Simulation Studies (p. 12), "Spatial patterns were simulated ... with source points generated from a homogeneous Poisson process and target points influenced by spatial interaction curves ..."

   This sounds to me as if the synthetic data were generated under exactly the same assumptions used for deriving the SIC curves proposed for analysis, is that correct? Would that not by construction give an advantage to the method proposed by the authors when comparing different analysis methods? It would be important to test the method also using other sources of synthetic data, e.g. starting from Bridson sampling or using a Gauss filter after the Poisson process.

   Response: This is an excellent point. Our primary simulation does generate data under the same conditional Poisson framework that SHADE assumes, which is standard for initial method evaluation—it allows us to isolate the effects of hierarchical structure, cell density, and sampling variation on estimation accuracy. Even under these ideal conditions, Section S4.4 demonstrates that hierarchical pooling substantially improves inference:

   > We found that on average, and across all spatial scales, the average RMSE of $\delta_{t_1 \to t_2}^{(m,p)}$ coefficients was lower for those estimated by the full hierarchical model than the model that fit images individually, most significantly so for closer-range coefficients (Table S2).

Section 3.1 now demonstrates that SHADE's performance relative to envelope tests depends on source cell density and the availability of multiple images for hierarchical pooling:

> First, when source density is high (regardless of target density), SHADE achieves excellent median power (100%) across all conditions, substantially outperforming envelope tests when target density is low (SHADE 100% vs. G-cross 77%, K-cross 73%). Abundant source cells provide strong conditioning information that hierarchical pooling can effectively leverage. Conversely, when source density is low, performance depends on whether multiple images are available: with 2–3 images per patient, median power reaches 100%; with only 1 image, power drops to 31% as limited conditioning information per image prevents effective pooling.

Robustness to spatial confounding: To address model misspecification, we added simulations testing SHADE's performance with unmeasured spatial heterogeneity (Section 3.1.1):

> We also tested SHADE's performance when the model is misspecified due to unmeasured spatial heterogeneity—specifically, discrete tissue compartments (e.g., tumor islands, stromal regions) that create baseline density differences independent of source-target interactions (Figure 5; Supplement Section S4.10). Results reveal regime-dependent bias: when both cell types are abundant, SHADE achieves perfect detection power but exhibits elevated Type I error rates (11.7–17.1%) and severely undercovers (43–52% vs. expected 95%), incorrectly attributing compartment effects to source-target interactions. When target density is low, wider credible bands provide partial robustness (82–93% coverage, 1.7–5.8% Type I error). These findings indicate that unmeasured spatial structure can produce substantial bias in high-density scenarios, suggesting the need for explicit compartment modeling or sensitivity analyses when such heterogeneity is suspected.

Minor points:

1. Introduction, "While our approach is not a direct extension of the multitype Gibbs points model ..., it is certainly inspired by it" (and similar statements in Methods): It would be very helpful to formally introduce these methods the paper is based on, possibly in a supplement, and then show direct comparisons on a suitable test problem. At the moment, it is not straight-forward to evaluate the extensions and modifications introduced by the authors.

   Response: This is an excellent suggestion that has substantially improved the manuscript's accessibility. We have added Supplement Section S1 ("Background: Multitype Gibbs Point Process Models and Relationship to SHADE") providing: (1) formal introduction to multitype Gibbs point process (MGPP) models and their symmetry constraint that prevents directional association modeling, (2) description of hierarchical Gibbs

models with type ordering [Högmander and Särkkä, 1999] that enable asymmetry via ordered dependencies, (3) SHADE's three key extensions (asymmetric SICs, flexible basis functions, multilevel Bayesian structure), and (4) comparison table (Table S1) contrasting MGPPs, hierarchical Gibbs models, and SHADE. The Introduction now references this supplemental material.

2. The introduction is quite long and contains sections that might be better placed in the discussion, such as specific comparison to other methods.

   Response: Thank you for this helpful organizational suggestion. The introduction has been substantially revised and streamlined. Method comparisons in the introduction establish the methodological gap and motivation, while detailed comparative analysis with results is included in the supplement (Section S5.7.3).

3. Methods (p. 7), "... assuming that ... follows an inhomogeneous Poisson point process": Could the authors indicate what that assumption implies in the context of typical applications of the new method (e.g., a Poisson process typically is based on a rare-events argument)? And would there be reasonable alternative choices for the probability distribution?

   Response: Thank you for this insightful question about model assumptions. We have expanded Section 2.1 to clarify what the inhomogeneous Poisson point process assumption allows and why it is the appropriate modeling choice:

   > This allows the expected density of target cells to vary flexibly across space as a function of source cell locations and covariates, enabling the framework to represent complex spatial patterns including clustering, repulsion, and distance-dependent associations. While alternative models exist (e.g., Cox processes and cluster processes), the inhomogeneous Poisson process provides the best balance of flexibility, interpretability, and computational feasibility for our application [Baddeley et al., 2015].

3 Response to Reviewer 3

We thank the reviewer for their thoughtful comments, which we believe have greatly improved this manuscript. Your feedback helped us clarify key methodological details and expand our comparisons with existing approaches. Below we have provided point-by-point responses to each comment. For easy reference, all manuscript changes are highlighted in blue, in both the responses here as well as the revised manuscript. **The blue text in the reply is a verbatim copy of the changes appearing in the paper**. We greatly appreciate the time and care you brought to reviewing this work!

1. How does the SIC compare to using functional data analysis (FDA) of the spatial curves that ones gets from K or G? For example, using basis functions that look similar in formulation to functional data analysis and functional principal component analysis?

   Response: Thank you for this excellent question about the relationship between SHADE and functional data analysis approaches. While both SHADE and FDA methods represent spatial relationships as smooth curves using basis function expansions, they differ fundamentally in what they model and how they leverage hierarchical structure. To address this, Supplement Section S5.7 now details:

   > SHADE directly models the underlying point process through the conditional intensity $\lambda(v \mid X_{A_1}, \ldots, X_{A_K})$ (Equation 2), leveraging the point-level likelihood for inference. In contrast, functional data analysis (FDA) approaches applied to spatial statistics (e.g., mFPCA of G-cross or K-cross functions) first compute per-image summary statistics, then analyze the resulting curves in a second stage—a two-stage procedure that does not propagate uncertainty from point pattern estimation into functional inference. While both methods can leverage hierarchical structure, SHADE's generative modeling framework directly integrates the spatial point process likelihood, enabling simultaneous estimation of spatial interactions and variance components across biological scales through partial pooling.

   To address this comparison, Section S5.7.3 now discusses:

   > To provide additional context for SHADE's results and explore complementary perspectives on group-level spatial organization, we compared SHADE's group-level SICs with functional data analysis (FDA) of marginal pairwise summary statistics. Specifically, we applied multilevel functional principal component analysis (mFPCA) [Wrobel et al., 2024] separately to CLR and DII patient groups, analyzing G-cross and L-cross functions computed for each tissue section.
   >
   > Unlike SHADE, which models conditional intensities adjusting for all cell types simultaneously within a generative point process framework, mFPCA decomposes functional variation in observed summary statistics across hierarchical levels (images nested within patients). This approach does not provide a probabilistic model of the underlying point process, but instead

characterizes dominant modes of variation in the empirical G-cross and L-cross curves. The resulting group-level mean functions with variability bands (mean $\pm$ 1 SD from the first functional principal component) provide a complementary view of pairwise spatial associations. Note that these bands represent descriptive variation captured by the first PC and do not have coverage properties analogous to confidence or credible intervals.

We have also updated the Discussion to contextualize these approaches:

> A key advantage of SHADE over traditional spatial summary statistics is its ability to formally model heterogeneity across biological scales through hierarchical partial pooling, enabling variance decomposition and more nuanced biological inference than post-hoc image-by-image comparisons.

2. It would be good to compare you approach to using FDA on the G or K statistics, as implemented in the R package mxfda(Wrobel et al. 2024). Additionally, should compare to not just G but also K as this has been used often in practice and has been reported to have better discrimination ability for detecting co-localization than G(Soupir et al. 2025).

   Response: Thank you for this constructive suggestion. We have added both G-cross and Ripley's L-cross function (transformation of the K-function) to our analysis. To address this, we implemented a comparison with mxfda [Wrobel et al., 2024] as described in Section S5.7.3 (detailed methodology in Supplement Section S5.7):

   > For instance, SHADE identified greater CTL clustering around tumor cells in DII patients across all distances (Figure 8), while G-cross mFPCA curves show the opposite pattern, with CLR patients exhibiting stronger CTL-tumor associations. This discrepancy likely reflects SHADE's multivariate adjustment: after controlling for other cell types, the conditional CTL-tumor interaction is stronger in DII, whereas marginal pairwise statistics capture different aspects of spatial organization. Similarly, SHADE detected greater granulocyte-TAM clustering in DII patients, but G-cross mFPCA shows minimal group differences for this interaction, suggesting that SHADE's hierarchical modeling and conditional framework reveals patterns that are subtle or absent in marginal analyses. Additionally, SHADE identified stronger CTL repulsion from CAFs at medium-long range in CLR patients, a pattern not evident in marginal G-cross analyses. These discrepancies highlight how multivariate adjustment and hierarchical pooling can reveal conditional dependencies that differ from marginal pairwise associations.

3. In the set-up of the model and approach in section 2.1, assume a inhomogeneous Poisson process. However, in the computational approximation using logistic regression you have a homogeneous Poisson process. In the logistic regression model fitting, how do you account for inhomogeneity? That is, is the analysis done within the tumor and

stroma compartments of the tissue as different cells could have different intensity within these two tissue domains. If this is not explicitly accounted for in the model, I would recommend that analysis be done by tissue domains to limit the issue of inhomogeneity.

Response: This is a great question about the relationship between model assumptions. To address this, Section 2.1 now clarifies that the inhomogeneous Poisson point process assumption allows:

> This allows the expected density of target cells to vary flexibly across space as a function of source cell locations and covariates, enabling the framework to represent complex spatial patterns including clustering, repulsion, and distance-dependent associations. While alternative models exist (e.g., Cox processes and cluster processes), the inhomogeneous Poisson process provides the best balance of flexibility, interpretability, and computational feasibility for our application [Baddeley et al., 2015].

Regarding the logistic regression approximation (now in Section 2.4):

> This approximation does not require the target cell process to be homogeneous—only the dummy points are placed homogeneously for computational convenience. Target cells are still modeled as an inhomogeneous Poisson process with spatial variation captured through distance-based SIC features $\boldsymbol{q}_{A_k}(v)$ and spatial covariates $\boldsymbol{z}(v)$ (Equation 2). In our colorectal cancer analysis, we demonstrate this flexibility by incorporating compartment indicators to control for tissue architecture (Section 4.4), showing how covariates can disentangle spatial interactions from architectural confounding.

4. In the simulation study, please state how many simulated datasets were generated for each scenario. I think you have 30 simulations per scenario but not sure. If this is the case that used only 30 simulations per scenario, why so few of simulations? Possible to have 100 per scenario to get more precise estimates of power and type I error rate to compare the 3 approaches? Also, state in the simulation set-up the range of cell abundances that assessed and why 39 simulated used to estimate envelopes for G.

   Response: Thank you for this question about study design. To address this, Section 3.1 now describes:

   > We simulated hierarchical point patterns (40 patients, 1–3 images per patient) with positive spatial interactions at multiple distance scales. We varied source cell density (15 vs. 150 cells per image) and target cell density (15 vs. 150 cells per image) across 50 replicates per condition.

   Each replication consists of multiple images (1–3 per patient), and hypothesis testing is performed at the image level, resulting in well over 100 image-level tests per scenario for precise power and Type I error estimates.

   For envelope test calibration (now in Supplement Section S4.5.4):

Global envelopes were constructed using 99 Monte Carlo simulations of point patterns generated according to complete spatial randomness (CSR). The rank parameters were calibrated in preliminary null scenario simulations to achieve proper Type I error control: `nrank = 10` for G-cross (yielding nominal significance level $\alpha = 10/100 = 0.10$) and `nrank = 8` for L-cross (yielding $\alpha = 8/100 = 0.08$). These nominal levels achieved empirical Type I error rates of 2.5–8.8% for G-cross and 1.3–6.7% for L-cross across different density regimes (Section S4.9).

5. Please have a "null" scenario and present the type I error rate. In Figure 5 only presenting the power which can't be interpreted without knowing the approaches control the type I error rate.

   Response: Thank you for emphasizing this important check for calibration. We evaluated Type I error rates using null scenario simulations where target cells are generated with no spatial dependence on source cells (all SIC coefficients set to zero). To address this, Section 3.1 now discusses:

   Coverage and type I error performance (Supplement Section S4.9) reveal adaptive calibration. When source density is high (favorable for detection), SHADE trades calibration for sensitivity (55% coverage, 100% power, <1% type I error). When both densities are low (unfavorable for detection), SHADE becomes appropriately conservative (94% coverage, 28% power, 4% type I error). This contrasts with SHADE Flat, which maintains poor coverage (73%) regardless of scenario and shows severely inflated type I error rates (28%). Median type I error rates for SHADE Hierarchical with 2–3 images are well-controlled (0.8–7.5%), comparable to envelope tests (G-cross: 2.5–8.8%; K-cross: 1.3–6.7%), though with higher variability (IQR up to 0.12), indicating occasional liberal inference under challenging conditions.

   Full results with figures are now presented in Supplement Section S4.9.

6. Please present computational time to fit the model. The colon cancer data (I believe) is based on a TMA. How would this method scale for computing on whole tissue slides with millions of cells?

   Response: Thank you for raising this important practical consideration. The colorectal cancer dataset does consist of tissue microarray (TMA) cores with hundreds to thousands of cells per image. To address computational scalability for larger datasets, we have added timing experiments (Supplement Section S4.8), now discussed in Section 2.5:

   Feature construction for each focal cell type B involves evaluating inter-cell distances between observed and dummy focal locations and all non-focal cells. This step scales as $\mathcal{O}(n_{\text{focal}} \times n_{\text{source}})$, which is quadratic in the total number of cells when the focal and source sets are of similar size. However, this operation is implemented using the optimized `crossdist` routine from

> `spatstat.geom`, which efficiently computes pairwise distances in compiled code. The resulting distance matrix is reused across all basis functions $\{\phi_p\}$ and source types $\{A_k\}$, so the dominant cost occurs only once per focal type.
>
> To assess the practical runtime implications, we performed a timing experiment varying the total number of cells from 5,000 to 250,000 using variational inference (Supplement, Section S4.8). Feature construction time scaled as $O(n^{1.46})$ (empirical exponent from log-log regression), while total model fitting time scaled as $O(n^{0.85})$ due to efficient distance matrix reuse. At 100,000 cells, total fitting time was approximately 36 seconds; at 250,000 cells, approximately 133 seconds. These benchmarks demonstrate that SHADE remains computationally tractable for large-scale multiplexed imaging studies.

Extrapolating from this empirical $O(n^{0.85})$ scaling to whole slide images with 1–2 million cells, we estimate total fitting times of 5–15 minutes per image using variational inference on a single core, which remains practical for large-scale studies. For MCMC inference, runtimes would be longer but could be accelerated via within-chain parallelization or GPU acceleration.

7. In the hierarchical model (section 2.2), please present where in the model you assess for differences by a factor/exposure (i.e., CLR and DII as used in the colon study).

   Response: Thank you for asking us to clarify this aspect of the model. To address this, Section 2.2 now describes:

   > When comparing spatial organization across biological groups (e.g., treatment responders vs. non-responders, different tumor subtypes), each group is modeled as a separate cohort with its own cohort-level parameters $\psi_{A_k}^{(g,p)}$. Differences between groups are then assessed by comparing the posterior distributions of these cohort-level parameters, as quantified through SICs (Equation 4) and their associated simultaneous credible bands.

   Specifically, for each source–target cell type pair, we compute the difference in cohort-level SICs between groups and examine whether the simultaneous credible bands around this difference exclude zero (e.g., Figure S21).

8. Please provide details on the analysis of the colon cancer data how you determined if the clustering was significant taking into account random chance? I don't see any information presented on how this would be done beyond just reference to figure that shows slightly different curves (Figure 6, Figure 9). In figure 9, looks like set arbitrary level at a score of 0.05.

   Response: Thank you for this important question about statistical inference. Under the inhomogeneous Poisson process model, the null hypothesis of "no spatial association" corresponds to SIC $= 0$ (i.e., the target cell intensity is conditionally independent of the source cell locations). Spatial clustering or repulsion is detected when the estimated SIC is significantly different from zero. To address this, Section 2.3 now describes:

> To quantify uncertainty in estimated SICs and assess statistical significance, we employ simultaneous 95% credible bands that account for multiple comparisons across the distance domain. Unlike pointwise credible intervals, simultaneous bands provide joint coverage across all distances within a specified range, offering stronger protection against false discoveries. Statistical significance can be assessed by examining whether the simultaneous band excludes zero over a distance range of interest. Full implementation details are provided in the Supplement (Section S4.1).
>
> In exploratory analyses involving many cell type pairs ($K(K-1)$ directed pairs for K cell types), we propose summary measures to facilitate prioritization: peak location and magnitude (identifying where the strongest interaction occurs), persistence over biologically relevant distance ranges (quantifying consistent associations within pre-specified intervals), and overall strength (integrating absolute effect sizes over significant regions). These measures can be computed across all source–target pairs and visualized as heatmaps for systematic comparison. Formal definitions are provided in the Supplement (Section S4.2).

When the simultaneous credible band excludes zero over a distance range, we conclude that the spatial association is statistically significant at that range. All SIC figures display simultaneous 95% credible bands computed using the method now detailed in Supplement Section S4.1.

Note: The 0.05 effect size threshold previously used to highlight substantive differences in log-intensity has been removed from the analysis.

9. G assumes homogeneity in the point process. Please look at results for G by tissue compartment (tumor, stroma, etc) to somewhat control for difference in intensity of different cell types by tissue domain.

   Response: This is an important point about controlling for spatial heterogeneity. The `mxfda` package only supports standard G-cross and not `Gcross.inhom`, the inhomogeneous version of G-cross from `spatstat`, limiting direct comparison of compartment-adjusted analyses. However, we have added an investigation of the impact of unmeasured compartment structure through simulation (Section 3.1.1; Supplement Section S4.10):

   > We also tested SHADE's performance when the model is misspecified due to unmeasured spatial heterogeneity—specifically, discrete tissue compartments (e.g., tumor islands, stromal regions) that create baseline density differences independent of source-target interactions (Figure 5; Supplement Section S4.10). Results reveal regime-dependent bias: when both cell types are abundant, SHADE achieves perfect detection power but exhibits elevated Type I error rates (11.7–17.1%) and severely undercovers (43–52% vs. expected 95%), incorrectly attributing compartment effects to source-target interactions. When target density is low, wider credible bands provide partial robustness (82–93% coverage, 1.7–5.8% Type I error). These findings

> indicate that unmeasured spatial structure can produce substantial bias in high-density scenarios, suggesting the need for explicit compartment modeling or sensitivity analyses when such heterogeneity is suspected.

This analysis informs interpretation when compartment structure may be present, indicating that SHADE's performance depends on the density regime and suggesting caution when both cell types are abundant without explicit compartment modeling.

10. What does the MAD presented in Figure 7 look like between CLR and DII tumors?

    Response: Thank you for this interesting follow-up question. We have added an analysis comparing MAD measures of heterogeneity between CLR and DII patient groups, now discussed in Section 4.3:

    > Granulocyte interactions with TAMs, vasculature, and hybrid E/M cells exhibited the highest heterogeneity, suggesting patient-specific or spatially localized microenvironmental factors (quantitative results in Supplement Figure S23).

11. A better literature review related to methods for co-localizations and those applied to mIF data in cancer should be in the introduction of the paper.

    Response: Thank you for this suggestion to improve the contextualization of our work. We have expanded the Introduction to include a more comprehensive review of spatial co-localization methods applied to multiplexed imaging data in cancer:

    > Standard pipelines for analyzing spatial cellular interactions in multiplexed imaging data typically compute second-order summary statistics, such as Ripley's K- and L-functions or the G-cross function, independently for each image to quantify tumor–immune interactions, infiltration patterns, or neighborhood structure [Barua et al., 2018, Vu et al., 2022, Canete et al., 2022, Samorodnitsky et al., 2024, Seal et al., 2024, Jing et al., 2025, Janeiro et al., 2024, Soupir et al., 2025]. These per-image summaries are then compared across groups or used as covariates in downstream analyses. Although effective for descriptive comparisons, this approach treats each image as an isolated point pattern and cannot share information across biological replicates during estimation. Multilevel models for replicated point patterns have been proposed [Bagchi and Illian, 2015, Lee et al., 2017, Myllymäki et al., 2014, Bell and Grunwald, 2004, Illian and Hendrichsen, 2010, Wrobel et al., 2024], but they typically model derived summaries via two-stage estimation or use parametric models fit via pseudolikelihood with random effects, rather than jointly estimating flexible interaction functions through full Bayesian multilevel inference at the point-process level.

    Additionally, Section S5.7.3 includes a detailed visual comparison with mFPCA applied to G-cross and L-cross functions, demonstrating the complementary strengths of SHADE and FDA methods for analyzing multiplexed imaging data.

Minor:

1. Figure 5: I can't seem to see the 20, 40, and 60 nm data. Also, add to caption what is hight and low levels.

   Response: Thank you for pointing out this visualization issue. Figure 4 (Detection power comparison) has been updated. The current caption is:

   > Detection power comparison across simulation conditions. Boxplots show the proportion of all images in which methods correctly identify non-zero spatial interactions by testing whether simultaneous credible bands (SHADE) or global envelopes (G-cross, K-cross) exclude zero anywhere in the 0–75 μm range. Results are stratified by source cell density (rows: the conditioning cell type) and target cell density (columns: the cell type being modeled), with number of images per patient (1, 2, or 3) shown on the x-axis. SHADE Hierarchical achieves highest power when source density is high, with performance when source density is low depending critically on having multiple images per patient available for hierarchical pooling (see main text).

References

A. Baddeley, E. Rubak, and R. Turner. *Spatial Point Patterns: Methodology and Applications with R*. CRC Press, Nov. 2015. ISBN 978-1-4822-1021-7. Google-Books-ID: rGbmCgAAQBAJ.

R. Bagchi and J. B. Illian. A method for analysing replicated point patterns in ecology. *Methods in Ecology and Evolution*, 6(4):482–490, 2015. ISSN 2041-210X. doi: 10.1111/2041-210X.12335.

S. Barua, P. Fang, A. Sharma, J. Fujimoto, I. Wistuba, A. U. K. Rao, and S. H. Lin. Spatial Interaction of Tumor Cells and Regulatory T cells Correlates with Survival in Non-Small Cell Lung Cancer. *Lung cancer (Amsterdam, Netherlands)*, 117:73–79, Mar. 2018. ISSN 0169-5002. doi: 10.1016/j.lungcan.2018.01.022.

M. L. Bell and G. K. Grunwald. Mixed models for the analysis of replicated spatial point patterns. *Biostatistics*, 5(4):633–648, Oct. 2004. ISSN 1465-4644. doi: 10.1093/biostatistics/kxh014.

N. P. Canete, S. S. Iyengar, J. T. Ormerod, H. Baharlou, A. N. Harman, and E. Patrick. spicyR: Spatial analysis of *in situ* cytometry data in R. *Bioinformatics*, 38(11):3099–3105, May 2022. ISSN 1367-4803, 1367-4811. doi: 10.1093/bioinformatics/btac268.

H. Högmander and A. Särkkä. Multitype Spatial Point Patterns with Hierarchical Interactions. *Biometrics*, 55(4):1051–1058, 1999. ISSN 1541-0420. doi: 10.1111/j.0006-341X.1999.01051.x. URL `https://onlinelibrary.wiley.com/doi/abs/10.1111/j.0006-341X.1999.01051.x`. _eprint: https://onlinelibrary.wiley.com/doi/pdf/10.1111/j.0006-341X.1999.01051.x.

J. B. Illian and D. K. Hendrichsen. Gibbs point process models with mixed effects. *Environmetrics*, 21(3-4):341–353, 2010. ISSN 1099-095X. doi: 10.1002/env.1008.

A. L. Janeiro, E. M. Wong, D. Jiménez-Sánchez, C. O. de Solorzano, M. D. Lozano, A. Teijeira, K. A. Schalper, I. Melero, and C. E. D. Andrea. Spatially resolved tissue imaging to analyze the tumor immune microenvironment: Beyond cell-type densities. *Journal for ImmunoTherapy of Cancer*, 12(5), May 2024. ISSN 2051-1426. doi: 10.1136/jitc-2023-008589.

S.-y. Jing, H.-q. Wang, P. Lin, J. Yuan, Z.-x. Tang, and H. Li. Quantifying and interpreting biologically meaningful spatial signatures within tumor microenvironments. *npj Precision Oncology*, 9(1):68, Mar. 2025. ISSN 2397-768X. doi: 10.1038/s41698-025-00857-1.

A. Lee, A. Särkkä, T. M. Madhyastha, and T. J. Grabowski. Characterizing cross-subject spatial interaction patterns in functional magnetic resonance imaging studies: A two-stage point-process model. *Biometrical journal. Biometrische Zeitschrift*, 59(6):1352–1381, Nov. 2017. ISSN 0323-3847. doi: 10.1002/bimj.201600212.

M. Myllymäki, A. Särkkä, and A. Vehtari. Hierarchical second-order analysis of replicated spatial point patterns with non-spatial covariates. *Spatial Statistics*, 8:104–121, May 2014. ISSN 2211-6753. doi: 10.1016/j.spasta.2013.07.006.

S. Samorodnitsky, K. Campbell, A. Ribas, and M. C. Wu. A Spatial Omnibus Test (SPOT) for Spatial Proteomic Data. *Bioinformatics*, 40(7):btae425, July 2024. ISSN 1367-4811. doi: 10.1093/bioinformatics/btae425.

S. Seal, B. Neelon, P. M. Angel, E. C. O'Quinn, E. Hill, T. Vu, D. Ghosh, A. S. Mehta, K. Wallace, and A. V. Alekseyenko. SpaceANOVA: Spatial Co-occurrence Analysis of Cell Types in Multiplex Imaging Data Using Point Process and Functional ANOVA. *Journal of Proteome Research*, 23(4):1131–1143, Apr. 2024. ISSN 1535-3893. doi: 10.1021/acs.jproteome.3c00462.

A. C. Soupir, I. V. Gadiyar, B. R. Helm, C. R. Harris, S. N. Vandekar, L. C. Peres, R. J. Coffey, J. Wrobel, S. Ma, and B. L. Fridley. Benchmarking Spatial Co-Localization Methods for Single-Cell Multiplex Imaging Data with Applications to High-Grade Serous Ovarian and Triple Negative Breast Cancer. *Statistics and Data Science in Imaging*, 2(1):2437947, 2025. ISSN 2997-9676. doi: 10.1080/29979676.2024.2437947.

T. Vu, J. Wrobel, B. G. Bitler, E. L. Schenk, K. R. Jordan, and D. Ghosh. SPF: A spatial and functional data analytic approach to cell imaging data. *PLOS Computational Biology*, 18(6):e1009486, June 2022. ISSN 1553-7358. doi: 10.1371/journal.pcbi.1009486.

J. Wrobel, A. C. Soupir, M. T. Hayes, L. C. Peres, T. Vu, A. Leroux, and B. L. Fridley. Mxfda: A comprehensive toolkit for functional data analysis of single-cell spatial data. *Bioinformatics Advances*, 4(1):vbae155, Jan. 2024. ISSN 2635-0041. doi: 10.1093/bioadv/vbae155.
